# Identification and Comparative Analysis of Long Non-coding RNAs in High- and Low-Fecundity Goat Ovaries During Estrus

**DOI:** 10.3389/fgene.2021.648158

**Published:** 2021-06-25

**Authors:** Yaokun Li, Xiangping Xu, Ming Deng, Xian Zou, Zhifeng Zhao, Sixiu Huang, Dewu Liu, Guangbin Liu

**Affiliations:** ^1^Guangdong Laboratory for Lingnan Modern Agriculture, College of Animal Science, South China Agricultural University, Guangzhou, China; ^2^State Key Laboratory of Livestock and Poultry Breeding, Institute of Animal Science, Guangdong Academy of Agricultural Sciences, Guangzhou, China

**Keywords:** long non-coding RNA, litter size, goats, reproduction, fertility, high-throughput nucleotide sequencing

## Abstract

The ovary is the most important reproductive organ in goats and directly affects the fecundity. Long non-coding RNAs (lncRNAs) are involved in the biological process of oocyte maturation. However, in the context of reproduction in goats, few studies have explored the regulation of lncRNAs. Therefore, we herein used the ovaries of high and low fecundity Leizhou black goats to identify differentially expressed lncRNAs (DElncRNAs) by high-throughput RNA sequencing; moreover, we analyzed the target genes of lncRNAs by functional annotation to explore the role of DElncRNAs in ovarian development. Twenty DElncRNAs were identified, of which six were significantly upregulated and 14 were significantly downregulated in high fecundity goats. Gene Ontology analyses suggested that MSTRG.3782 positively influences the expression of the corresponding gene *API5*, exerting regulative effects on the development of follicles, through which litter size might show variations. The target gene KRR1 of ENSCHIT00000001883 is significantly enriched in cell components, and ENSCHIT00000001883 may regulate cell growth and thus affect follicular development. Further, as per Kyoto Encyclopedia of Genes and Genomes pathway analyses, MSTRG.2938 was found to be significantly enriched, and we speculate that MSTRG.2938 could regulate ribosomal biogenesis in the pre-snoRNP complex as well as cell transformation in eukaryotes. Quantitative real-time PCR results were consistent with sequencing data. To conclude, our research results indicate that some lncRNAs play a key role in regulating follicle development and cell growth during goat’ s ovarian development.

## Introduction

Litter size is influenced not only by nutrition levels and environment but also by inheritance (Cui et al., 2009). The ovary is the most important organ for the normal reproductive function of goats. It secretes estrogen to maintain sexual characteristics and cyclical reproductive activity; further, oocytes and ovulation have a major impact on the fertility of goats (Barnett et al., 2006; Zhao et al., 2015). Studies have shown that the ovulation rate of goats is linked to high productivity (Pramod et al., 2013). lncRNAs play a chief role in reproduction-related processes in animals, but very limited information is available on the functions of lncRNAs in goats. In particular, in the context of reproduction in goats, few studies have explored the regulation of lncRNAs (Xing et al., 2014). Long non-coding RNAs are non-coding RNA transcripts of >200 nucleotides in length; they have a complex structure and lack the ability to code proteins (Jarroux et al., 2017). They can regulate gene expression and protein function to perform biological functions. Studies have reported that lncRNAs can regulate reproductive processes, such as ovarian development and maturation in female animals (Li et al., 2015; Ling et al., 2017; Liu et al., 2020). Therefore, it is crucial to study their role by exploring the function of key target genes.

High-throughput RNA sequencing and functional analyses have been used to elucidate the reproductive function of lncRNAs that were identified to be differentially expressed between the ovaries of multiparous and uniparous Anhui white goats; TCONS_00136407, TCONS_00146968, and TCONS_00320849, for example, were suggested to participate in oocyte meiosis (Ling et al., 2017). Using the same method to study the function of differentially expressed lncRNAs (DElncRNAs) in Chuanzhong black goats, ENSCHIT00000005909 and ENSCHIT00000005910 were suggested to regulate the viability and proliferation of keratinocyte-derived cells by influencing *IL1R2* (interleukin 1 receptor type II) thereby affecting ovarian function (Bouckenheimer et al., 2018). Leizhou black goat is a special local goat breed in southern China, which shows excellent adaptability to the living circumstance with high humidity and high temperature, and using high-throughput sequencing and bioinformatics analysis can help us to explore the novel functional DElncRNAs in the ovaries of goats.

Litter size is one of the most important economic traits in goat production, determining the benefit of farming enterprises. To provide a theoretical basis for goat breeding and improve the production efficiency of goat industry, it is vital to conduct in-depth research on the mechanisms regulating litter size. We herein screened DElncRNAs between the ovaries of high and low fecundity Leizhou black goats and predicted the target genes of DElncRNAs. In addition, Gene Ontology (GO) and Kyoto Encyclopedia of Genes and Genomes (KEGG) pathway analyses were used to analyze the function of target genes. Our results not only enrich the transcriptomic data of the goat ovary but also provide a theoretical basis for combining molecular breeding and conventional breeding technologies.

## Materials and Methods

### Ethics Statement

All study protocols were approved by the Ethics Committee for the Care and Use of Laboratory Animals at the South China Agricultural University (permit no.: SYXK-2014-0136). Further, all experiments were performed in accordance with the guidelines of the South China Agricultural University.

### Animals and Sample Collection

Seven healthy female Leizhou black goats (age, 3.5–4.5 years) were divided into high and low fecundity groups. The litter size of high-fecundity group (*n* = 3) and low-fecundity group (*n* = 4) were more than one and only one, respectively. Meanwhile, all of the samples in this study were from goats with three parity records of litter size. The female goats were injected with 0.1 mg cloprostenol to induce estrus (Perera et al., 1978; Martemucci and D’Alessandro, 2011; Fierro et al., 2013). The goats were kept under observation to determine whether they were in heat (bleating, searching for the male goat, frequent urination, hyperemia, edema, contraction of the vulva, and vaginal mucus discharge). The basis of estrus was the female goat shaking their tail, standing, and accepting to mate with the male goat (Taylor, 1978; Mekuriaw et al., 2016). The ovaries were collected within 24 h of estrus. The selected goats were killed and dissected, and both whole ovaries from each goat were collected immediately. The intact ovaries were collected and washed with 75% alcohol thrice. Then they were soaked into phosphate buffered saline. After the collection of the ovary, the ligaments and attached tissues were trimmed off under surgical anatomy microscope, ovarian follicles were isolated from the ovary, and the isolated ovarian tissue was frozen in liquid nitrogen and stored at –80°C.

### Total RNA Isolation, cDNA Library Construction, and Transcriptome Sequencing

After thoroughly grinding the ovarian tissue, total RNA was extracted using TRIzol (Invitrogen, Carlsbad, CA, United States). NanoDrop ND-2000 was used to measure RNA concentration (Thermo Science, Wilmington, DE, United States). RNA integrity was assessed by denaturing agarose gel electrophoresis. Further, the cDNA library was constructed using 3 μl of total RNA from each sample, and double-terminal sequencing was performed on the HiSeq X-TEN sequencing platform by Shanghai Parsons Biotech Co., Ltd.

### Quality Control of Raw Sequences

We used Cutadapt to remove reads with an average quality score below Q20. The Q20 value referred to the error probability of 1% for the identified bases in the process of base recognition. The reference genome index (GCF_001704415.1_ARS1_genomic^1^) was established by Bowtie 2, and the filtered reads were compared with the reference genome using TopHat 2. If the mismatch between the reads and the reference genome sequence was within 2, we considered the alignment to be successful (Kim et al., 2013).

### Assembly and Novel lncRNA Prediction

According to the TopHat 2 results, StringTie was used for transcript assembly, and candidate lncRNAs were then selected based on the splicing results and structural features of lncRNAs. The screening conditions to identify lncRNAs were as follows: (1) transcripts with low expression levels, low credible single exon transcripts, and exon numbers < 2 were filtered out and (2) transcripts < 200 bp in length were excluded (Trapnell et al., 2010; Cabili et al., 2011). Moreover, Coding-Non-Coding-Index v2 (Barnett et al., 2006), Coding Potential Calculator (0.9-r2; Sun et al., 2013), Pfam Scan v1.3 (Punta et al., 2012), and phylogenetic codon substitution frequency (v20121028; Lin et al., 2011) were used for coding potential analyses. Transcripts without coding potential comprised the candidate set of lncRNAs. lncRNA expression at the transcription level was analyzed with StringTie. DESeq was used to analyze the expression of lncRNAs; the screening conditions were | Log2FoldChange| > 1 and *P* < 0.05 (Love et al., 2014). The ggplot 2 software package was used to construct a volcano map of DElncRNAs, and the pheatmap software package was used to perform clustering according to the expression level of same lncRNAs in different samples and that of different lncRNAs in the same sample. Distance was calculated with the Euclidean method and clustering was performed using hierarchical agglomerative clustering (Wang et al., 2010).

### Target Gene Prediction

To explore the functions of lncRNAs, we predicted the target genes of DElncRNAs. Because the reliability of the analysis results is not high when the sample number is small, the function of trans-regulation can not be predicted. We searched the genes 100 kb downstream and upstream of lncRNAs and analyzed their functions.

### GO and KEGG Pathway Analyses for Target Genes of DElncRNAs

GO analysis was performed with the predicted target genes using DAVID^2^. Furthermore, we used the KEGG database to analyze the potential functions of these genes in pathways^3^ (Dennis et al., 2003; Han et al., 2012). A hypergeometric test was applied to discover the significant enrichment of GO terms and KEGG pathways so as to determine the main biological functions of differentially expressed genes (Wang et al., 2020; Huang et al., 2007). *P* < 0.05 indicated statistical significance.

### Quantitative Real-Time PCR (qRT-PCR) for DElncRNAs

Total RNA (1 μg) was first reverse-transcribed using an RT Reagent Kit with gDNA Eraser (Takara, Dalian, China), according to manufacturer instructions. qRT-PCR was performed on a StepOnePlus Real-Time PCR System (Life Technologies, United StatesA), as per the standard protocol, using TB Green Fast qPCR Mix (Takara, Dalian, China). Primer Premier 5 used in primer design. Capra hircus β-actin served as the endogenous control for mRNA and lncRNA expression analyses.

## Results

### Sequencing Data Quality Control

The raw reads from the high and low fecundity groups were analyzed for quality control before further analyses. The Q30 value for each sample exceeded 93% (Table 1). Within the mapped reads, >85% of total reads were mapped to the reference genome without any mismatch (Table 2), indicating that the sequencing data was of high quality and suitable for subsequent analyses.

**TABLE 1:** Quality control of RNA-seq data.

Sample	Clean reads (bp)	Clean reads (%)	Q30 (bp)	Q30 (%)
LL_ovarian1	102,568,128	99.71	14,469,811,737	93.78
LL_ovarian2	105,050,222	99.76	14,876,252,498	94.18
LL_ovarian3	101,304,042	99.66	14,193,808,086	93.09
LL_ovarian4	106,886,182	99.74	15,093,669,895	93.89
LH_ovarian1	101,039,510	99.51	14,381,870,205	94.43
LH_ovarian2	104,636,964	99.36	14,844,532,299	93.97
LH_ovarian3	101,631,476	99.59	14,245,966,824	93.07

*Q30 value represents the error probability of 0.1% for the identified bases in the process of base recognition; LL_ovarian1–LL_ovarian4, low fecundity goats; LH_ovarian1–LH_ovarian3, high fecundity goats.*

**TABLE 2:** Statistics of the mapping result.

Sample	Total-mapped(bp)	Multiol-mapped(bp)	Uniquely-mapped(bp)
LL_ovarian1	87,722,258(85.53%)	3,401,453(3.88%)	84,320,805(96.12%)
LL_ovarian2	94,045,775(89.52%)	2,863,389(3.04%)	91,182,386(96.96%)
LL_ovarian3	88,004,353(86.87%)	2,413,301(2.74%)	85,591,052(97.26%)
LL_ovarian4	95,760,551(89.59%)	2,719,333(2.84%)	93,041,218(97.16%)
LH_ovarian1	90,985,423(90.05%)	2,200,033(2.42%)	88,785,390(97.58%)
LH_ovarian2	93,646,857(89.50%)	2,601,986(2.78%)	91,044,871(97.22%)
LH_ovarian3	89,517,832(88.08%)	2,404,052(2.69%)	87,113,780(97.31%)

*Multiol-Mapped, the total number of sequences aligned to multiple positions; Uniquely mapped number of sequences with unique alignment positions on the reference sequence.*

### Screening and Validation of DElncRNAs

**FIGURE 1:**
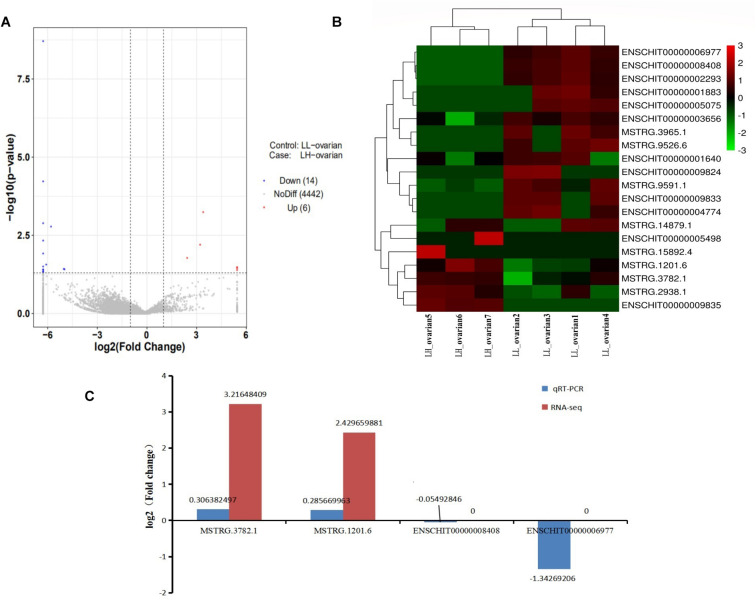
Analysis and validation of DElncRNAs in RNA-seq libraries. **(A)** Volcano map in analyzing DElncRNAs between high fecundity group and low fecundity group. The red plot represented up-regulated expression in high fecundity group; the blue plot represented down-regulated expression in high fecundity group. **(B)** Hierarchical clustering analysis of lncRNA expression profiles from libraries with 20 DElncRNAs. Data were expressed as FPKM. Red: relatively high expression; Green: relatively low expression. **(C)** qRT-PCR results pertaining to DElncRNAs were compared with RNA-seq data. Red: RNA-seq; blue: qRT-PCR.

Of 4,462 lncRNAs, 20 were differentially expressed between the high and low fecundity groups. Compared with the low fecundity group, six lncRNAs were upregulated and 14 were downregulated in the high fecundity group (*P* < 0.05; Figure 1A). From the heatmap analysis, the expression level of the same lncRNA in the same group was essentially the same, indicating that there was little difference between the samples in the same group. Four and three samples belonging to the low and high fecundity groups, respectively, were clustered together, indicating that lncRNA expression patterns in the groups were different (Figure 1B). Six DElncRNAs were randomly selected for qRT-PCR to verify the reliability of RNA sequencing data. qRT-PCR results were fundamentally consistent with sequencing results, confirming that the sequencing data had high reliability (Figure 1C).

### GO Analyses for Target Genes of lncRNAs

#### GO Analyses for Target Genes of All lncRNAs

**FIGURE 2:**
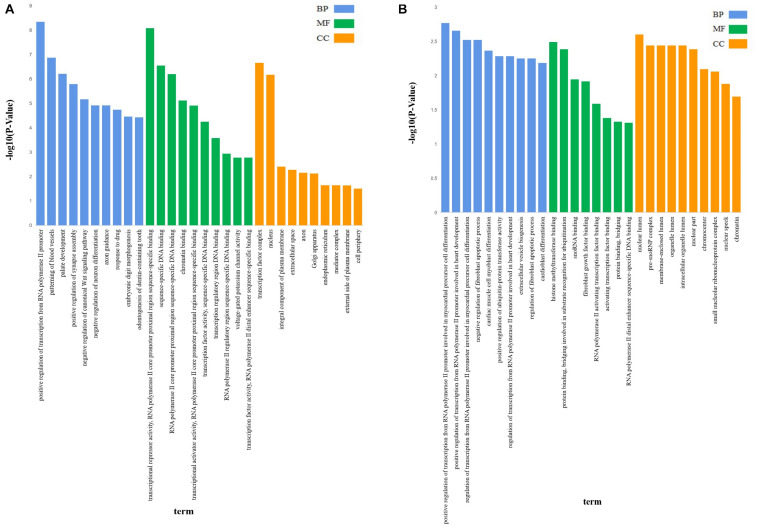
The GO analysis of target genes of lncRNAs. GO analysis of lncRNA-target genes according to biological process (BP), cell component (CC), and molecular function (MF); **(A)** The GO analysis of target genes of all lncRNAs, *X*-axis was *P-*value(-log10), *Y*-axis was the GO term. **(B)** The GO analysis of target genes of DElncRNAs, X-axis was *P*-value(-log10), *Y*-axis was the GO term.

The 4,462 lncRNAs corresponded to 2,870 genes, of which DElncRNAs corresponded to 19 target genes. To explore the biological function of lncRNAs involved in regulating litter size, we performed GO and KEGG pathway analysis to identify the functions of target genes. GO analyses revealed diverse biological functions, such as positive regulation of transcription from RNA polymerase II promoter, patterning of blood vessels, and palate development, and multiple target genes were involved, such as *CTNNB1* (encoding catenin beta-1), *WNT5A*, and *EDN1* (endothelin-1). Transcription factor complex, nucleus, and integral component of plasma membrane were the top three terms significantly enriched in the cellular component, whereas transcriptional repressor activity, RNA polymerase II core promoter proximal region sequence-specific binding, sequence-specific DNA binding and RNA polymerase II core promoter proximal region sequence-specific DNA binding were the top three terms significantly enriched in the molecular function (*P* < 0.05; Table 3 and Figure 2A). *ZNF536* and *SALL1* (sal-like 1) are noted to be involved in these functions.

**TABLE 3:** Top 10 significantly enriched Gene Ontology (GO) terms of target genes of all long non-coding RNAs (lncRNAs).

GO ID	GO name	Observed gene count	*P*
**Molecular function**			
GO:0001078	Transcriptional repressor activity, RNA polymerase II core promoter proximal region sequence-specific binding	25	8.11923E-09
GO:0043565	Sequence-specific DNA binding	50	2.81848E-07
GO:0000978	RNA polymerase II core promoter proximal region sequence-specific DNA binding	44	6.27029E-07
GO:0003682	Chromatin binding	44	7.63455E-06
GO:0001077	Transcriptional activator activity, RNA polymerase II core promoter proximal region sequence-specific binding	32	1.22538E-05
GO:0003700	Transcription factor activity, sequence-specific DNA binding	55	5.63408E-05
GO:0044212	Transcription regulatory region DNA binding	21	0.000261987
GO:0000977	RNA polymerase II regulatory region sequence-specific DNA binding	10	0.001142393
GO:0005249	Voltage-gated potassium channel activity	10	0.001653743
GO:0003705	Transcription factor activity, RNA polymerase II distal enhancer sequence-specific binding	7	0.001653743
**Biological process**			
GO:0045944	Positive regulation of transcription from RNA polymerase II promoter	79	4.46E-09
GO:0001569	Patterning of blood vessels	13	1.33E-07
GO:0060021	Palate development	20	6.08E-07
GO:0051965	Positive regulation of synapse assembly	16	1.60E-06
GO:0090090	Negative regulation of canonical Wnt signaling pathway	20	6.79359E-06
GO:0045665	Negative regulation of neuron differentiation	14	1.19474E-05
GO:0007411	Axon guidance	20	1.20265E-05
GO:0042493	Response to drug	18	1.83209E-05
GO:0042733	Embryonic digit morphogenesis	14	3.44706E-05
GO:0042475	Odontogenesis of dentin-containing tooth	13	3.71271E-05
**Cellular component**			
GO:0005667	Transcription factor complex	32	2.15852E-07
GO:0005634	Nucleus	232	6.64207E-07
GO:0005887	Integral component of plasma membrane	74	0.003866795
GO:0005615	Extracellular space	81	0.005325092
GO:0030424	Axon	16	0.006989932
GO:0005794	Golgi apparatus	50	0.007490282
GO:0005783	Endoplasmic reticulum	45	0.022593322
GO:0016592	Mediator complex	7	0.022693577
GO:0009897	External side of plasma membrane	20	0.022811136
GO:0071944	Cell periphery	5	0.031220242

#### GO Analyses for Target Genes of DElncRNAs

GO analysis revealed that 47 terms were significantly enriched between the high and low fecundity groups; the target genes involved were *IER2* (immediate early response protein 2), *TBXT*, *API5* (encoding apoptosis inhibitor 5), *KRR1*, *ARRDC4* (arrestin domain containing 4), *NOP56* (encoding nucleolar protein 56), and *OIP5* (encoding OPA-interacting protein 5) (*P* < 0.05). The target gene *API5* of MSTRG.3782 participated in 14 GO terms, including nuclear lumen, negative regulation of fibroblast apoptotic process, and regulation of fibroblast apoptotic process. The target gene *NOP56* of MSTRG.2938 participated in 13 GO terms, including nuclear lumen, histone methyltransferase binding, and pre-snoRNP complex. Further, we classified the function of the target genes into the three major GO categories of biological process, cellular component, and molecular function. Positive regulation of transcription from RNA polymerase II promoter involved in myocardial precursor cell differentiation, positive regulation of transcription from RNA polymerase II promoter involved in heart development, regulation of transcription from RNA polymerase II promoter involved in myocardial precursor cell differentiation were the top three abundant terms in the biological process category (*P* < 0.05). In the cellular component category, nuclear lumen, pre-snoRNP complex, and membrane-enclosed lumen were the top three abundant terms, whereas in the molecular function category, histone methyltransferase binding, protein binding, bridging involved in substrate recognition for ubiquitination, and snoRNA binding were the top three abundant terms (*P* < 0.05; Table 4 and Figure 2B).

**TABLE 4:** Top 10 significantly enriched Gene Ontology (GO) terms of target genes of differentially expressed long non-coding RNAs (DElncRNAs).

GO ID	GO name	Genes	*P*
**Molecular function**			
GO:1990226	Histone methyltransferase binding	NOP56	0.0032
GO:1990756	Protein binding, bridging involved in substrate recognition for ubiquitination	ARRDC4	0.0041
GO:0030515	snoRNA binding	NOP56	0.0113
GO:0017134	Fibroblast growth factor binding	API5	0.0121
GO:0001102	RNA polymerase II activating transcription factor binding	TBXT	0.0257
GO:0033613	Activating transcription factor binding	TBXT	0.0415
GO:0030674	Protein binding, bridging	ARRDC4	0.047
GO:0000980	RNA polymerase II distal enhancer sequence-specific DNA binding	TBXT	0.0486
GO:0060090	Molecular adaptor activity	ARRDC4	0.0563
GO:0001158	Enhancer sequence-specific DNA binding	TBXT	0.0602
**Biological process**			
GO:0003257	Positive regulation of transcription from RNA polymerase II promoter involved in myocardial precursor cell differentiation	TBXT	0.0017
GO:1901228	Positive regulation of transcription from RNA polymerase II promoter involved in heart development	TBXT	0.0022
GO:0003256	Regulation of transcription from RNA polymerase II promoter involved in myocardial precursor cell differentiation	TBXT	0.003
GO:2000270	Negative regulation of fibroblast apoptotic process	API5	0.003
GO:0060379	Cardiac muscle cell myoblast differentiation	TBXT	0.0043
GO:0051443	Positive regulation of ubiquitin-protein transferase activity	ARRDC4	0.0052
GO:1901213	Regulation of transcription from RNA polymerase II promoter involved in heart development	TBXT	0.0052
GO:0140112	Extracellular vesicle biogenesis	ARRDC4	0.0056
GO:2000269	Regulation of fibroblast apoptotic process	API5	0.0056
GO:0010002	Cardioblast differentiation	TBXT	0.0065
**Cellular component**			
GO:0031981	Nuclear lumen	IER2, TBXT, API5, KRR1, NOP56, OIP5	0.0025
GO:0070761	Pre-snoRNP complex	NOP56	0.0036
GO:0031974	Membrane-enclosed lumen	IER2, TBXT, API5, KRR1, NOP56, OIP5	0.0036
GO:0043233	Organelle lumen	IER2, TBXT, API5, KRR1, NOP56, OIP5	0.0036
GO:0070013	Intracellular organelle lumen	IER2, TBXT, API5, KRR1, NOP56, OIP5	0.0036
GO:0044428	Nuclear part	IER2, TBXT, API5, KRR1, NOP56, OIP5	0.0041
GO:0010369	Chromocenter	OIP5	0.008
GO:0005732	Small nucleolar ribonucleoprotein complex	NOP56	0.0087
GO:0016607	Nuclear speck	API5, OIP5	0.0131
GO:0000785	Chromatin	TBXT, OIP5	0.0202

### KEGG Pathway Analyses for Target Genes of lncRNAs

#### KEGG Pathway Analyses for Target Genes of All lncRNAs

**FIGURE 3:**
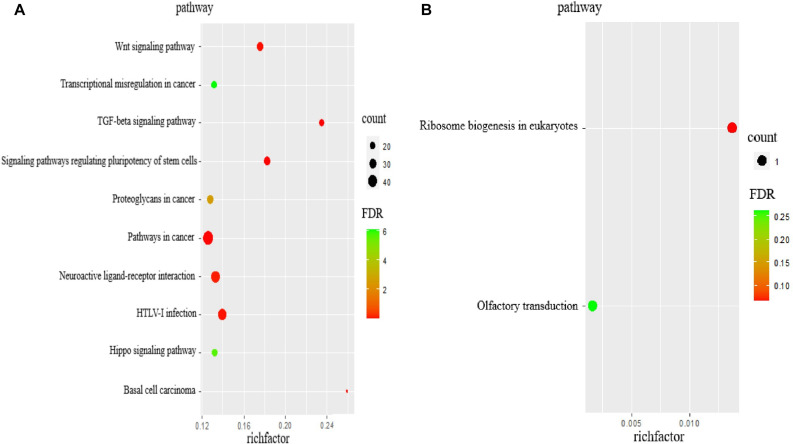
The KEGG analysis of target genes of lncRNAs. **(A)** KEGG pathway analyses of target genes of all lncRNAs. KEGG enrichment was measured by rich factor, FDR and the number of genes enriched on this pathway. **(B)** KEGG pathway analyses of target genes of DElncRNAs. KEGG enrichment was measured by rich factor, FDR and the number of genes enriched on this pathway.

As per KEGG pathway analysis, 20 pathways were significantly enriched (*P* < 0.05). The top 10 pathways were primarily associated with transforming growth factor-beta (TGF-β), signaling pathways regulating pluripotency of stem cells, pathways in cancer, basal cell carcinoma, Wnt signaling pathway, HTLV-I infection, neuroactive ligand-receptor interaction, proteoglycans in cancer, Hippo signaling pathway, and transcriptional misregulation in cancer (Table 5 and Figure 3A). The target genes included *CTNNB1*, *WNT5A*, and *TGF-*β*2*, among others. The target gene *WNT5A* of ENSCHIG00000000774 was involved in seven signaling pathways, such as the Wnt signaling pathway, basal cell carcinoma, and HTLV-I infection.

**TABLE 5:** Top 10 significantly enriched Kyoto Encyclopedia of Genes and Genomes (KEGG) pathways of target genes of all long non-coding RNAs.

KEGG pathway	Number of genes	*P*
TGF-beta signaling pathway	19	3.48E-06
Signaling pathways regulating pluripotency of stem cells	25	7.62E-06
Pathways in cancer	49	1.28E-05
Basal cell carcinoma	14	3.25E-05
Wnt signaling pathway	23	3.61E-05
HTLV-I infection	34	4.89E-05
Neuroactive ligand-receptor interaction	35	9.47E-05
Proteoglycans in cancer	25	0.00199537
Hippo signaling pathway	20	0.004450684
Transcriptional misregulation in cancer	20	0.004786637

#### KEGG Pathway Analyses for Target Genes of DElncRNAs

According to KEGG pathway enrichment analyses, the target genes involved ribosomal biogenesis in eukaryotes and olfactory transduction pathways, of which only the former showed significant enrichment (*P* < 0.05). The target gene *NOP56* of MSTRG.2938 was involved in this pathway (Table 6 and Figure 3B).

**TABLE 6:** Top significantly enriched Kyoto Encyclopedia of Genes and Genomes (KEGG) pathways of target genes of differentially expressed long non-coding RNAs.

KEGG pathway	Number of genes	*P*
Ribosome biogenesis in eukaryotes	1	0.033754056
Olfactory transduction	1	0.261408061

## Discussion

The elucidation of mechanisms regulating litter size can provide a theoretical basis for breeding technologies in goats. Therefore, in this study, we used the ovaries of high and low fecundity Leizhou black goats to identify DElncRNAs by high-throughput RNA sequencing; moreover, we analyzed the target genes of lncRNAs to explore the role of DElncRNAs in ovarian development.

We herein identified enriched terms and signaling pathways; subsequently, we analyzed them as well as pertinent target genes involved in the regulation of reproduction. Fibroblasts are the main cellular component of loose connective tissue (Cai et al., 2012; Yeung et al., 2013). Carcinoma-associated fibroblasts evidently regulate the development of epithelial ovarian cancer by affecting the proliferation, apoptosis, migration, and invasive activity of ovarian cancer cells (Zhang et al., 2011). Fibroblast growth factor (FGFs) is involved in follicular development and follicular atresia (Costa et al., 2009; Miyoshi et al., 2010; Asgari et al., 2015; Coticchio et al., 2015). The expression of FGFs related to follicular development changes with the development of follicles. FGFs need to bind to distinct receptors to physiologically function (Basu et al., 2014). Apoptosis inhibitor 5, which is encoded by *API5*, is involved in regulating the cell cycle. Further, it promotes DNA synthesis and cell cycle G1/S transition, and regulates cell growth, proliferation, and apoptosis (Garcia-Jove Navarro et al., 2013). *API5* plays an important role in the termination of diapause and early embryonic development of Artemia sinica (Zhang et al., 2017). Through GO analysis, we found that the target gene *API5* of MSTRG.3782 was involved in the regulation of fibroblast apoptotic process and FGF binding. MSTRG.3782 was significantly upregulated in the high fecundity group. Accordingly, we hypothesized that lncRNA participates in cell growth, thereby affecting follicular development.

The nucleus is the main repository of genetic information in eukaryotic cells, and the site of DNA replication and transcription; it consequently controls genetic and metabolic activities (Lynch and Marinov, 2017). Chromosomes are the most important structures in the nucleus and carry hereditary information (Zetterström, 2008). A study found that OPA-interacting protein 5 (*OIP5*) was enriched in centrosomes during the G1 phase of the cell cycle and mediated the regulation of cell division (Naetar et al., 2007). In addition, *OIP5* reportedly has a fundamental role in maintaining the structure and function of centrosomes/centromeres (Fujita et al., 2007). *KRR1* encodes proteins present in early 90 S precursor particles of the small ribosomal subunit, and its locus has been implied to contribute to the development of polycystic ovary syndrome (Gromadka and Rytka, 2000; Zheng et al., 2014; Pau et al., 2017). In this study, we found that the target gene *OIP5* of MSTRG.1201 and the target gene *KRR1* of ENSCHIT00000001883 were significantly enriched in the cellular component of nuclear lumen, chromatin, organelle lumen, intracellular organelle lumen, among others. In the high fecundity group, MSTRG.1201 was significantly upregulated and ENSCHIT00000001883 was significantly downregulated. Therefore, we believe that MSTRG.1201 and ENSCHIT00000001883 affect follicular development by regulating cell division.

Mature snoRNP particles are composed of a series of small nucleolar RNA and core proteins. snoRNPs regulate the processing and modification of pre-rRNA and play an important role in ribosomal biogenesis (Richard and Kiss, 2006). Nucleolar protein 56 (encoded by *Nop56*) is involved in the synthesis of snoRNP as a core protein (Lykke-Andersen et al., 2018). Furthermore, an increase in the ribosome biosynthesis rate can promote the expression of the proto-oncogene *C-myc* and enhance the proliferative ability of cancer cells (Tomczak et al., 2015). *C-myc* encodes a transcription factor with a direct role in controlling translation (Ruggero, 2009). *Nol5a/Nop56* may be a critical gene involved in Myc-mediated oncogenic transformation (Cowling et al., 2014). According to our GO and KEGG pathway analyses, MSTRG.2938 was significantly upregulated in the high fecundity group, and its target gene *NOP56* was involved in ribosomal biogenesis in eukaryotes and the pre-snoRNP complex. We thus speculate that MSTRG.2938 regulates ribosomal biogenesis in the pre-snoRNP complex as well as cell transformation in eukaryotes. However, the specific mechanism of regulation of each lncRNA remains to be further investigated.

According to GO analyses, the target genes [Guanine Nucleotide Binding Protein, alpha 13(*GNA13*), Mothers against decapentaplegic homolog 2 (*SMAD*2), and Fibronectin Leucine Rich Transmembrane Protein 2 (*FLRT2*)] of all lncRNAs were mainly enriched in positive regulation of transcription from RNA polymerase II promoters, patterning of blood vessels, palate development, and positive regulation of synapse assembly. RNA polymerase II plays a pivotal role in the transcription of protein-encoding genes in all eukaryotic cells (Bernecky et al., 2016). *GNA13* participates in regulating cell movement and developmental angiogenesis (Offermanns et al., 1997). Moreover, *SMAD2* overexpression has been reported to repair secondary cleft palate by increasing apoptosis of medial edge epithelial cells in the TGF-β3 pathway (Miyazono et al., 2018). We thus report that these genes play a major role in maintaining the healthy growth of goats.

The Wnt signaling pathway and its downstream effectors not only regulate physiological processes such as cell growth and differentiation, cell migration, and genetic material stability but are also important for cancer progression, including for regulating tumor growth, cell senescence, and cell death. The Wnt/β-catenin signaling pathway is involved in various important processes, such as the regulation of embryo development, cell proliferation, and cell migration (Nusse and Clevers, 2017; Peng et al., 2017; Steinhart and Angers, 2018). β-Catenin is an essential structural component of cadherin-based adherens junctions and is a key component of Wnt/β-catenin signal transduction. Aberrant expression of *CTNNB1* and *WNT5A* has been observed to affect cell proliferation and lead to cancer occurrence. A mutation in *CTNNB1* is one of the many causes of β-catenin degradation (Kim et al., 2018). The Wnt/CTNNB1 pathway is a pivotal signaling pathway that regulates steroid production (Abedini et al., 2016). *WNT5A* is a highly evolved conservative non-classical Wnt ligand, which is required for normal ovarian follicle development (Abedini et al., 2016). *WNT5A* is differentially expressed during the development of mouse follicles, and it can significantly inhibit steroid production in atretic follicles (Lapointe and Boerboom, 2011; Abedini et al., 2016; Kumawat and Gosens, 2016). By blocking the function of FSH (follicle-stimulating hormone) and luteinizing protein, *WNT5A* can induce the down-regulation of *CTNNB1* and cAMP-response element binding protein (CREB), thus affecting follicle development and gonadotropin reactivity (Abedini et al., 2015). We found that the target gene *CTNNB1* of ENSCHIG00000000641 is one of the two signaling pathway members of Wnt and proteoglycans in cancer. ENSCHIG00000000641 was downregulated in the high fecundity group. Further, the target gene *WNT5A* of ENSCHIG00000000774 participated in the negative regulation of canonical Wnt signaling pathway terms and the Wnt signaling pathway. ENSCHIG00000000774 was also downregulated in the high fecundity group. Thus, we believe that ENSCHIG00000000641 and ENSCHIG00000000774 affect follicular development by regulating cell proliferation and steroid production, respectively.

The TGF-β superfamily participates in many physiological activities in mammals via autocrine and paracrine pathways, and TGF-β is mainly produced locally in the ovary. It has been reported that *TGF-*β*1* can promote the growth of mice follicles. Both TGF*-*β and activin A have proliferative action and cytodifferentiative action on granulosa cells (Liu et al., 1999). As a member of the transforming growth factor β family, TGF*-*β2 also plays an important role in the growth and development of follicles. TGF*-*β2 is located in follicular membrane cells and luteal cells and regulates the production of inhibitors and activins in granulosa cells and luteal cells (Knight and Glister, 2003). *Smad2/Smad3* are key molecules in the TGF-β/Smad signaling pathway that regulate ovarian growth and development and maintain ovarian function (Coutts et al., 2008; AlMegbel and Shuler, 2020). *Smad2* and *Smad3* can maintain normal fertility in women, and further support the Smad2/3 pathway in the ovary to participate in the regulation of signals produced by oocytes, which plays an important role in the coordination of ovulation (Li et al., 2008). In this study, we found that *TGF-*β*2*, *TGF-*β*R2*, and *Smad2* participated in the TGF-β signaling pathway, and they were regulated by ENSCHIG00000000886, ENSCHIG00000000609, and ENSCHIG00000002761, respectively. We accordingly speculate that these lncRNAs regulate follicle development, but the specific mechanism needs to be further studied.

To conclude, we found that target genes of all lncRNAs were mainly involved in protein transcription and played a role in maintaining the healthy growth of animals. In addition, the TGF-β and Wnt signaling pathways were found to be related to reproduction in animals. Based on functional analyses of target genes of DElncRNAs, fibroblast apoptotic process, FGF binding, pre-snoRNP complex, and ribosomal biogenesis in eukaryotes were associated with reproduction in goats. Our data improves the current understanding of the transcriptome of goats and provides valuable information for functional genomics resources and biological studies; moreover, we believe that our results are of great significance for in-depth studies of candidate lncRNAs in breeding techniques.

## Data Availability Statement

The datasets presented in this study can be found in online repositories. The names of the repository/repositories and accession number(s) can be found below: NCBI (accession: PRJNA728366).

## Ethics Statement

The animal study was reviewed and approved by all study protocols were approved by the Ethics Committee for the Care and Use of Laboratory Animals at the South China Agricultural University (permit no. SYXK-2014-0136). Further, all experiments were performed in accordance with the guidelines of the South China Agricultural University.

## Author Contributions

YL: conceptualization, methodology, writing-reviewing, and editing. XX: data curation, writing-original draft preparation, software, and validation. MD: conceptualization. DL: visualization and investigation. GL: supervision. XZ: investigation. ZZ: investigation. All authors contributed to the article and approved the submitted version.

## Conflict of Interest

The authors declare that the research was conducted in the absence of any commercial or financial relationships that could be construed as a potential conflict of interest.

## Footnotes

1 http://www.ensembl.org/

2 https://david.ncifcrf.gov

3 http://www.genome.jp/kegg/

## Supplementary Material

Supplementary File 1 The DElncRNAs in the ovary of high fecundity goats and low fecundity goats. (XLSX)

Supplementary File 2 lncRNA primers sequences used for the qRT-PCR. (XLSX)

Supplementary File 3 Protein-coding genes detected 100-kb upstream and downstream of the DElncRNAs. (XLSX)

Supplementary File 4 GO enrichment analysis of target genes co-located with the all lncRNAs. (XLSX)

Supplementary File 5 GO enrichment analysis of target genes co-located with the DElncRNAs. (XLSX)

Supplementary File 6 KEGG enrichment analysis of target genes co-located with the all lncRNAs. (XLSX)

Supplementary File 7 KEGG enrichment analysis of target genes co-located with the DElncRNAs. (XLSX)

Supplementary File 8 Sequences of the DElncRNAs. (DOC)
