# Supplementary material for: Identification and Comparative Analysis of Long Non-coding RNAs in High- and Low-Fecundity Goat Ovaries During Estrus
Source: Front Genet. 2021 Jun 25;12:648158. doi: 10.3389/fgene.2021.648158 (PMC8267794; doi:10.3389/fgene.2021.648158)
Supplement: Supplementary File 8 — Sequences of the DElncRNAs. (DOC) [file Table_8.DOC]

>ENSCHIT00000009831 gene=ENSCHIG00000007243

TGCATTTCTATTACCCTGGATAGTCTAGATGGCACTTTTTAGATCTCACGTTTTGTTTGGCTTCATCTGGGCCAGTTAGTTTTGTTCTCACCCCTACTTACAAACTGTCAAAACACGCCATCCATTTCATAACTGTGTTCTATATAAAAAAAGAACAACTAGACCATTTTCATCTCAGTTGCTGGGGAAGAATTCCAGTGGAATGGACCTAACAACACAAGGGAACTGGAAGGGAAGCTTCAGGCAGGCTGACTCAGATACCCAGGCTCCTTTCTGCAGTATCTCTGGAGACAACAGCAGCAGACGGACAGCAGTCGGAAGGCATGTTGCAGCTCACATTGTAACTCGCTGGAAGAGGGTGATGGCAAAGCAAATGGAAGTCAAAGGGCACAGAACTCAAATCTGCAGCTTCCTGGAAAATAAATTACTTCTAGCCTAGAAGGTACTCCTGTCTTCAGTTTCTTTTCCTTTTAATATACTCAGAATGCCTACCACCACATTGTTTTTTCTGAAAAAAATTCCACTACATATCATGAGTTGACAAGAAAATCTCCAATGGCTCCAAGCTGACTGGAGGGTGGTTCTTTAATTTGCACTAAGGGCCCTCCCCCAGTAGTTCTATCCTCCCCCAAATAAGCCCTTTCCCTGACAAAACTGGCCCATCCTCTGTCCCTGGAACAATGCCTGTCCCCACTGGAACATTCTGGAACACTCTATTTCCTTTCTTGGAGAATTATAATCCTTGCTTTCCAGCTAATGTTCACCAAACACTTGCTACATACTAAATGCCTATTTTATTTCATTCTCATTACAGTTCTATGGAGAATGAGTTATTATTATCCTAACTTCACATTTCAGAATACTGAGGCTTAGATAGGTTATGAACCTACAGTCACATAGCCAGTAAGTGGGCAAGCAGAGATTTGAACTGAGGTCTTTACTTTCAGGGCCAAACTCTTTACCACTGTGGTGCAATGAGC

AGGACTATCTGATGGTGACCCCAGAGCTCAGGCAGCGTCACCTGCATGATAAACAGAGCAGGTGAACACCTTTTCCCCAGCAGAACTGTACTTCATATCAGTTATCCACAGTGTTGGTGACTTCCTCCCTCAGAAGAAGAAACTGAAACTTCCCATGGGTCTGAAAGTCTCTAGTCCAGAAGGTGACATTCATAAATGCCTATAAACAGCTGGGACACCAAACTGAACTGTTTGGAACTAAGCAGCCTGAGTATTCCTCCAATGCCAAGGATACTTTCTCATTCTCTCACTATGGTAAGGCAGAATGACAAGAGAACTCAAACCCACGCTGCAGGCTCACAAAGCCCAAGCCCAGTACACATCTCAGTGGGACAATGGGAACACTGGGGGAACTGTGCCTTCTTTTCTACTCCTGACTGAGCCGTGTTAATTTTCCCATTTCCTTAGTAAAATCTATTCCTTAACACACGCAGGGTGAAGTTGTATGATCAGTCTTGAATCCTGGTACATGGTGATTGAAGACCTAGATGGAATGTGCTGGACACAGCCACCACCTATGATGCCTTTTGGCATAAATGTTAACCACTCTTTTACAGTTGAGCTAAAGTCCCACTTATTCTACAAAGAAGTTCCTGATTGTCCTTAGTGAAGGGGGATCCTTGGAGCTCCAAGGATCCTATAGCAGAGTTTATCCTCTGGCCTCTACACAAAGCACATCTCTGATAAGTTATTGGTTACGGCATCTTTTCCACTAACAAATGTAGACATGAGAGAGATTCTAAGTTTACAGATCATCGCAGTAAAGTGAGTCCTACAGGTTGTTCTGGTTTCCCAGGGCACAGAAAAGTCATGTTTACACCATACTGTAATCTATTAAGCATGCAATAGCATTAGGTCTTAAAAAATAATGCATATACGTTAATTAAAAACACTTGATTGCTAAAAAAATGCTAAGTGTCATCTGAGACTTCAGTTAGTTGAAATCTTTTTGGCAATAGAAGCATTGAAAATCACTGATCACAGATCACCATAATGTATTAATAATAATGAAAAAGTTTGAATTTTGTGAGAATTACTAAAATGTGACACAGATACATAAAGTGAGCAAATGTTGTTATAAAAATAGTGCCAGTAGACTTGTTTGATGCAGGGTTGGTCACAAACCTTCAGTATGTAAAAAACCTAATATCTGTGAAGTCAATAAAACAAGATATGCTTGTGTGCATTTAGAATCCAATGAAATTTTCATCTTCGCAAAGGTAGGGGAGGGACCCTATAAATTTAGTACCTGTAACAGGCCCAGTGGCAATCCTAGATACAACAAAGATTAATGCCTATTTGCTG

>ENSCHIT00000005075 gene=ENSCHIG00000003824

AGACAGGAGCACAGTTGGGAGCTGGGTGGAGCTCTGACTCCAGGAACATTTCTCAGGCTAGAGGTTGTTCCCTTAGCTACCTGCCTCGGGCCAACAACCTGAAAATGAATCCTTGTTACAGCTGTGCATCTGGCCTATGTGGATGGAGGCACTGCTGGCAAATGTGAGATTTCTGAGGACACAGATTGGGAATTCCAGGATCTGGTCAGATTGGAAGTGCAATGGGCTTCATTTGATGGGAGCTAGCAGTCCTAGAACTACCCCCTTTGAATTGTATCTTGGAAAAACTGGGATATAAAGAAGCTCTTCTTCTCAAGTCACTGATGGTTTAAAACAATTTAATGATTTACACTTATGGGTAAAATTAAAACATTTTTCTTCTCTCTCTGCCTTGTCCCTCCAAAGTTGGAGACACTTGGGTTCTGAACAGCCTTATCAAGGTAAAAAAGACTGTGTCCCAACATATACAGAAATCTCAAAGTATACTGGGGAGCTCTAAAAGAAAAATCCACCCAACTGATGTCAGGCCTATGGCATCTATCATGTTTCACTGAGTATTACATTCTAGTTTTATTAATTAAGGTCTGTATTTACTGACTCACTTCTGAGTTAGTTCCTTGTGGTTATGTTACATTGCTAAGAGGTTTAATTAAGTTATTAAAAAGGACACTCTGAGTTTGTTTCTAAAGTTTATCCCAGTAACCAGTCTTTAAATAAAGATCAGATGCTCCAGGATCTACAACCAGAAGATTAATAGCAGGCTATAGTCATTTAACAAACTCAGTCAGATGACCTGGAAATATCACTGGGCTATACCTAATTAAGTTCTTGACTTAAGGTCTTACTTGTGATTTTACTCTTACTGCTAACTATATTGTTTTCTATATTGCCTGTTTCAAGAAGTGTTGTCCCTTACATTATCAAGTCTGTGATGGAGCCACTGATATAATGATGGTGTGCAGTTCCATATAAAATCCATAATTATAATGGTGTGACTCTGTATATGAAAAGAAGCAAGAGGGAATATTCTCCTGGACCAAGAGACTAGTGAGACAGGTGGGTCCAGAGATTTTGCTACTCACAGGGCCTAATACAGTAACAGCACATTGAGTGGCCTATCAGTGAAATCTTCAACAGACCTGGTAATGAGCTTTCCCAGCACTGTGGGACAAGATGGTCATGAAACGCCCCCAAACCATGGTCAAACTCATGGCCATAAAGGGGCCCTGTGACTGGAAATTGGCCCTTGCCATCTGCCTCTACAAAGATTAAATCATGGACACTACAGCTGCTGACCTTCGGCATCCTCTGAAAGGAGTTCAGGGTGAAAATCAGAGATTAGACACTTGCTCTGTGCTCTGAAAAAAAAATGGACAAAACAAGCCTTTGAATAGTCAGATATTTTCAGGTAAAAAAATTTTTGAGCCCAAATTCTTGCATTTTCTCATACCTAGAAAAACTCTATTTACAACTGTTAATAGTAACAACTACTTTGGCTTATATGTTAATACCACAATAAGAGGCTAAAGCCTACTTGGACAAACTTAGACAACTAGCTATGACCATAGTGAAAGTGAAAGTCGCTCAGTCGTGTCCAACTGTTTGCAACCCCGTAGACTAACAGCCCGTGGAATTCTCTAGGCCAGAATACTGGAGTGGGTAACCTATAAGTCTCCCTAAAACAGCTGTATACATAAGGGTTCAGACTCATTCTTCTAAAAAGAAATGGTAAGATTTATTTTGTCTGTTAAGGTTCAGGTTGTCAGTCTTTCAACTACTTCTAACTGGGTTTGTTATACCCACATTCAAAATGTATATGAACATGCCAAATGGCTCCATTCTTATAAATAGGGCAGCCCAAGTGTTGACTATATCTAAACCAGGATCAAAGTTACTAGCAAATGTAACCTGATTCTTACTCTTGCTTGGACTGCTGATTACAGTAATTCTCTTACTGATT

TTTGGTCTTTGCCTTTTAATCTACTTGTCTAGTTTGTTCTTTCTGAAGGATACAACAGCTTCCCATACAAAGTAACGGTGATGCAGAGATTCTGGTCACTCTTCAAAACTGGACTCCCCTGATGTTCCACTCTGATCGTAGATATAATCTTGGTTTACAAGCTCTCCTTGGTGAAAAACTATCTTGACTGTAATACTTGTAATCATTCTGATTTTGCTGTTTTTTCCTTACATCTGTAACTGTGTAATAAGATTTGTTTCTAACCATCTGAAAACATTTAAATTACAAATGATTGTTCATGCTCCTACAATTTCTACTGCCTCCTCTAGCTATTACTTGGGATCCTTGGATCAGAAACCCTAAATAAAGGGGCAATGAGAATACATTGCTTCAACAACTTAGGGTCTGCCCTCCTCACCTGCAGGAAGCAGTTACTGACAGATTTCTCTACCCCTTTCTCCTCAGCAAAAATATTCTCCTAAAAGAAAAAGGGGAGAAGGAGAGAGTTAATTTAGGCAGGTTGATAAGCAGTCCAAGCCTAAGGCCTCTTTATGGAGGAGGTACACATTCTTTCTTTTTCACATTTTTCTGCCCTGGACAAATAGGCCTTTGTAGGCAGCAGTATCTCTTGTTCAAGGACATGCCTCTTTTAGAGCTTTG

>MSTRG.2938.1 gene=MSTRG.2938

CGCGGTGCATGCTGGGGCCCGCGCGGCCCGAGGGGCTGCGGAGCCGCCGGCTAGCCCACCATGTCCACCCACCTGCGGCAGCAGAAGGCTGATCTCCTCCACCTCCTTCAGCGCCAGAAGCGCGTAGCCGACCGCGTGCTCGAAAAGCACGTGCAGCAGCACCTGAAAAGGGAGCCGGGATACCAGAGATCTCAGTTCCTGACCAGAGATTGAGCCAGGGCAATGAAAGTCCAGAATGTTAACTACTAGGCCACCAGCGGATTCCTATATCAGCTTCAATTATGAAATAAAAGGGTGCATTGACTAGAAGGAAAGAATGTCTATGTGAAAAATAAAGATTAAATGTCTCCTTTCCTAGGATTCCAATTCCAGTCCTCCTTCAATGATAAGACTCCCTTCCCAGGTGCCAAGGCCATACTGACTCATTGTGTATGTGCTGATCTATTTCTTTTTTTTTTGAAACCTCGAAGGAATGTATCCCTGATTTGTTTAATGTTCTTTGTTCTGATAAGATATAAAACTGTGCTGAAAACCAGGATTCTCTGGAGCAGCTTCTCAGAGTTATCTAAGAGGCTGTCTTCTGGGATATATTCTTCAGTTTGGCTCAAATAAATCCTTTTCTCTTCCTATTATAGATTGTTTATTGATTATTTTTGAGACACCGTGGAACACAGAAGAGTCATTAAAATGTGTGTTCATGTATCCACATGAGAAAGTC

>ENSCHIT00000001883 gene=ENSCHIG00000001359

TTGTTACTTCCCCTAACCCACGCAGAGAGCACATAAAGATTTTATTTCTTTGAAGGAAAAAGGAGAAAGAAATATGTTTGCTCTAACATGACGCAGAAATCAATGATGATGCTGTCTTAACTTGCTGAGCTTGTAGAAAGAGAAACGCTAGTAAAATTTAATACTCTGAATCATCAAAGGATAAATGGAAGAAGACATCTTTATAAATAGGTGCCAACAGGAGACAGAAAAGGCAAGAAAAAATGAAGCAGAGAAAAGGAAGTTTCAGAGGCGAGCAGCAGGCACAGTGTCCGAAGGGACAGAGATCAACCGCGGCGGTGTGGGATAAAGCAACAACATCGGCGGTAACAGAGAGGTCGCAGTTGACTTTCAGAAAGATGGACATGTCTGGAGCCCATAAAGCTCAACAAAATCCTGTTTGGCACTGCCTTCAACCCTGCAGGATGACCTTCCTCATGAAGCTAAAGAGAGGAGCCAAGGCTTAAGCATTTCTTCCTTGAGAGAACCAGAGGAAACCTGAGAAGCTATACTCATGGAGCATCTCCTAACTGGTGTTATCTTGTAGACTTCATTGAGAGTTATATACCCTTTAAGCTAAATGTGATAACGTATAATTCATATAAAATCTATGTGTTTCAGTCTCATCCTCTCACATTTTCAAATCCAGTCTTCTATAGCCCTAATTCATACTCCAGATGTCTACCTTTCTACTATGTCATCCACTGCATTACTGCTGAGCTCCAGCTCACACAGACACACACACACACACACCATAAACAAGAGAGATTCAGAGTCGGCGTATGAGAAACATTTTAGTATGACCTAAAGAAAACATACGTGCATAAATATGAAATGAACAAACGTCTGGCATACAAAATGTATTCCAGCATATATGATGTATTTTGATAATTGCTATTTTATTTTCAATGCTAATTTTCATAAACTGATTAGTTCATCAAATAAGAGGTCTCATCCTACAGTTCAAATCCATTTTGCCGTACATTCAATTTTCAGATTGGACCCTGGAATCCATTAGTGTACAGTCAATGAAATTCTTTACAGAGTTATCTCCTTTTCTGGCCTACCATTTAACTTTGTTTTCTTTATAAGAATGTCGCATGAAATAATTGTGCCAAGTATTTTCTTTCTTTCTCAATTAATCTCTGAATGTGAAAGGAAAATATATATCCTCACTTGCAAGTAAAATTAAGAGTGACGGGCTTCTCTGAGAAAGATAAGTGTATATATGCATGTACATGAACACAGATACATAGAGAAGGATGTAATTACAGTAAATATAGATTTTTCTTCTTCTTTACTTAGTTTCTCAAGAGGCAGGTTAAGTGGTCTGGTATTCCCATCTCTTTCAGAATTTTCCACAGTTTATTGTGATCCACACAGTCAAAGGCTTTGGCATAGTCAATAAAGCAGAAATAGACGTTTTTCTGGAACTCTCTTGCTTTTTCCATGATCCAGTGGATGTTGGCAATTTGATCTCTGGTTCCTCTGCCTTTTCTAAAACCAGTTTGAACATCAAGGAGTTCACGGTTCATGTATTACTGAAGCCTGGCTTGGAGAATTTTGAGCATTACTTTACTAGCGTGTGAGATGAGTGCAATTGTCTGGTAGTTTGAGCATTCTTTTGCATTGCCTTTCTCTGGGATTGGAATGAAAACTGACCTTTTCCAGTCCTGTGGCCACTGCTGAGTTTTCCCAATTTGCTGGCATATTGAGTGCAGCACTTTCACAGCATCATCTTTCAGGATTTGAAACAGCTCAAATGGAATTCCATCACCTCTACTAGCTTTGTTCGTAGTGATGCTTTCTAAGGCCCACTTGACTTCACATTCCAAGATGTCTGGCTCTAGATTAGTGATCACATCATCATGATTATCTGGGTCGTGAAGATCTTTTTTGTACAGTTCTTCCATGTATTCTTGCCACCTCTTCTTAATATCTTCTGCTTCTGTTAGGTCCAGACCATTTCTGATCTTTATCGAGCCCATCTTTGCATGAAATGTTCCCTTGGTATCTCTAATTTTCTTGAAGAGATCTCTAGTCTTTCCCATTCTGTTGTTTTCCTCTATTTCTTTGCATTGATCCCTGAAGAAGGCTTTCTTATCTCTTCTTGCTATTCTTCGGAACTCTGCATTCAGATGCTTATATCTTTCCTTTTCTCCTTTGCTTTTCACCTCTCTTCTTTTCACGGCTATTTGTAAGGCTCCCCAAACAGACATTTTGCTTTTTTGCATTTCTTTTCCATGGGGATGGTCTTGATCCCTGTCTCCTTTACAATGTCACGAACCTCATTCCATAGTTCATCAGGCACTCTATCTGTCAGATCTAAGCCCTTAAATCTATTTCTCACTTCCACTGTATAATCATAAGGGATTTGATTTAGGTCATACCTGAATGGTCTAGCGGTCTTCCCTACTTTCTTCAATTTGAGTCTGAATTTGGTAATAAGGAGTTCATGATCTGAGCCACAGTCAGCTCCTGGTCTTGTTTTTGTTGACTGTATAGAGCTTCTCCATCTTTGGCTGCAAAGAATATAATCAATCTGATTTCAGTGTTGACCATCTGGTGACGTCCATGTGTAGAGTCTTCTCTTGTGT

>MSTRG.9591.1 gene=MSTRG.9591

GGAGGAGCCTGTAGGATTAGTGCGGTGTCCAGGGTGGGGATGCTGGTGCTCCCCTGGACCCTCATTAGATCTAGACAGTCGGGAGTAGAGCCTGAGAATCTGCATTTGTAATAAGGTTGGGGCTGATGCTGCTGGTCTTCAGATCACACTTTTAGTAGCAAGAACGAACATAGATCAGGATTCCCACATGCTGTGTATCAGCCT

>ENSCHIT00000009824 gene=ENSCHIG00000007235

CTGGCCTGTTGTCCCCCCGCAGTAGCTGCTGAATGGATGAGTGTTAGGGGCCAAGTGATCCCTGATATATGGGAGGGAAAGAGGAACCCTGGGCCTAGAGAGGTCGGCCAGCCAGAGCCAGGAGTAGGTTGTTCTATCCGGTCGCCACCACCGTCACGTGATCCTGAAAGCCTTCCTCGCGCCTGGAATTCCTTCCACCCCTACTTCTCAAAGATGCTGCGCACACTAAGACGGAACGCGAAAGTTCTTGGAGGGGTCCTGCGGCTGCTCCAGCTCCGGGACCTGGCTTCGCAAGGACTCCATTTCGAACTCGGCTCCGGCTCGGTCTTCCCTCGACGCCCCCAGCGCCGCCCGGGCCCCCTCCATCCTGTGCGCAGAGGAGCGGAGAGACGCCGAGCGGGACGCTCCTGGGCCCAGCGCTCTGGACTGTGCTGATTTTTGAAAGTTATGGCTTGGGCGGAGAGCGGTTGGCCAAGCCTGTAGAAATCCTGGGACCTCAGTAAAATCAGCGGGTCCAAAGGAAACGGGCCTGCTTGTGGAAAGGAGCAGAGCTGAGTATCTGGGGAAGGAGCGGGACACCTGAGAGGTCGCCCCCAAGGCAGAAGTCGCAGGGACACCTACTCTGGAGGCCCGACTCCCACTCTGCGAACTCAATAAAGACTGAGAGATTAATAAGCTTGAAAATGTGTATTTTCACCGGGACCAGTAATCTAAATACCGTTAAAAATATATAGATTCCTGGCGGACTCCACCTAGACCTACCGAATCTCATAAGGGGATAAAGAGGCCAATTTTCAACAAGCGCCCCAGGACTTCCCAGGTGGTGCTAGTGGTAAAGAACTTGTCTGTCAAGGCAGGAGACATAAGAGACTCTAGTCTGATCCCTGGGTCGGGAAGATCCCCTGGAGTTGCTGCTAAGTCACTTCAGTCGTGTCCGACTTTGTGCGACCCCATAGATGGCAGCCCACCAGGCTCCCCTGTCCTGGGATTCTCCAGGCAAGAACACTGGAGTGGGTTGCCATTTCCTTCTCCAGTGCATGAAAGTGAAAAGTGAAAGTGGTCGCTCAGTCGTGTCCGACTCTTAGCGACCCCATGGGCTGCAGCCCACTAGACTCCTTCGTCCATGGGATTTTCCAGGCGAGAGTATTGGAGTGGGGCGCCATCGCCTTCTCGGCAAATTATGCTAAGAAATTTAAATAAAAATTTTTGAAGAGGAAAAAACCTTGTATTCTACAAGTCCCCTGCCACAATTATCTAAGCTTTTCAGTAATGCAGCAGCAGTATCATTTCAATGCTTTGTCAAACTAATGGCTCCAGAATACTATAAGCCAGGCTTCAGAGCAAAATTAAGATTTTAGTTTTACTAGTTGAGCATTTTGTAAGAAATCACAACATAAGTCTAATATAAACATATGTGCCTTGAAGACACTGCTATCAGATAAAATGTAAACAGTTTCAAATATGAATTAAACAAAATTATCCCCAGTGAGTGTGGGGATACTAAGAAAAGTGTCCGTTTCTATAAACATCAAAAAGTATTTTTAATTAATTCTTCACTGCATATAAATGTCTGCTCTATTTGATAACTGCAGCAAATACAATAAACACAGTTATCAAACATCAGCCTGAACCATAAAGAAATCTTTATAAACAAAGAAATAATAAAATTCCTAATAATCT

GAGATACAGAAGTGAGAAGGATTAGCAACTGAGTATCTGTCTATTGGTATTTTTAAAATTTTACTTAAGAGACCTCAAGGTTAACATTTTAAAATAATTTGGCTACATTACCATGTAGTATTTAATAAGTTTATTTGGGCTTATACCATGAAAGTATCATCTGACTAATCAAAGAATTTTAAAATAAAAATACTAGTAATTTTGATTTCT

GTCAACATATCTGGCCCAAACCATGTTTTTCAAAAGAAACTGGATACAACAGCACCCACTGAAAAACAAAGGTGGGGAGAGGGGCTGTAATTAATAAGGAACCAATATGAACCAAATCTCCCTACAACACAAGAAATTATTAATCAAATGTACTTCAGATGTACTTTATCTTTGGGGTTTCTGGGTATGTCCTCACTATAAAAATAACCTAAGTTAAACAACTGCTCCGTTACACTGATACTGGATCCAAATCAAAGAATCAAGCCGACTCCTGAAGATATCCTCTGTGCCAGGCAATGGGATTACAAAGAGGCATTTAATTAAGACAAGCTCTCTACCCTCAAGGAGCCCATCACTCTCCTAGAGAAGAGAGGGTATTAAATACAGTAACAACAAAAGGGGCATGATCCAAGTGCCAAATGAATGTCTATAATGGATGGAATACTATCAAATAGTAGTACTGCAGGGATTCTAGACTAGAATGACCACAGGACTGAAGAGGTTAGAGGAGGCTTTGTGCAATACATAGAAGCCATTATTTGAAGGAAAGACAGGCC

>MSTRG.3782.1 gene=MSTRG.3782

GTCGACGCTAGAGGAGGAGGAGCCGAGGTGGTGCCGCCGGCTGACAAGCGGCTTCCTCCGCCTCTGTTTGGGCGAAGCGGAAGCGATGCCTTCGGCCCTGTCCCTGTTTGGCTTCAGGCCTGAGCGAGACTGGACTAGTGGAAGTCGACAGGAGGACTGCACTGGGCATGGAGGAGAATGCAAGAGGACGCATCTTTGCCAGCCGGTGCTTTCCCGCCTGCCTCTGCCTTCCAGGCTTCTCCTCCTGAAGGAGCAGACAACGGGAGGACAAGCAGGAGGCTGACGAGGCCCAAGAAGGATCGTTAATACCACATCTGCCAGAGGCTGTATCTGTTCCTGAGGGAGAATGGACAACCCTTCTTGCTTTGAAGCTAAAACAGGGACAATCAGGAATGGATGGTTCATGTCATCTTCCTTCCTTTTTTTTTTCTGTTGGCATCTCTGAATACAACAGGCTTATTTTTCAGGTTCTTAGTGTTACATGCTCAGCTCAGTGAACGCAGAAGGGTGACTTAGAAAGGAATAGGATAAGTTGTTTTCCCAGCAGTTCAAGTTCCCCAAGCTAACGCAAAAAAAGTAACCTGAAAGAGCTATGATACTCTTCAATTATTACTTAAAATCAAGTATAACACAGTTTCTGCCTAATCTCATATTTCTTGTACCCAAACGGAAACGCTCACCATAATTAAAGTTGCTCAAATTGCTGCTATATTTCCCACTAGGAGGATTATAAATCTGCCTGGCATCCCTTTTTGGTCCTGCTGAAGATTTCTTGGGAGGTGAACCTGAAGTTGTATCTTCACTGGCTCTCTTTTGCCTATAATGGTAGGAAGAGCAGCAATGAAAAAAAAATTCTTTTATATCAGAGGCTAGCAATAAATTTATCATATTAGTCACAAAAGACACCCTAAAGATGATAGTGACATCAGAAAAAAGGAAATAACATCTGATGTCATCAAATTAACTCTGCTTCGGTTATGCAGACATTGTCACAAGGGCATTTACAAGGCTCTAAAACAATCTGAAATATGTAGCTTCATAGATTATACACAAGTGACTCATTTCATAGAACTTACCAAAAAATTCTTATGCCACAGCAATACAGTGGTTTGAAAATTCCAGAGTTGTAGTCCACCAGTTTATTTTATAAACACTAGATTAGATTTTCTATGTAAGTCATGCTTCTAATAGCTATATTTTAACATCTTATTAAGACTCATTTAATAATGTACCATTTTGTACAGGAAATGCAATAATCTGAATTCAGACTTTTCATATTTATAAATCAAATATTAGAACACTTTAATCCCTTTTAAATATGTTTCTTACCCAAGTTCAACCTTCTGTACAGGCTTCCAGGATAATGTTACTGTGCTCTTATAAGAAGGAGGAATATGGAAGAGATCCTGAAGAAACAAAATGAAGAGATTTCTGAAGTATTTGGTGGTGTGGCACGTGGCAGTAAAGATAGGAAATGGGAAGACACATCTTTAATCTTAGAAGGAGAATAACTTATCCACTTGCTTTTGTGCTAGTGACTAAAACAAGAACCTAGGGAAAACTGCATGAGAAAGTAAACAAGGACAATTTGGTTTGCAAATGACATACATTACCTAATTAATGTCATTTTGTGCACATCACTTAAGAAACTGCAGCTAACTTTTTTCAAGTGTCGTTTCAGGTTACAGGACAAAATATCAATTTTTATCAACTTCAATAAAATCTCCAAGTGGTTTCTTTTGCACAGATG

CAGTCACTAAGGTGCTTTAAGGAAAATATAATGGTGCAATCTGCATTTGCTATTGCAGATTTTTAAGTGGTTCAATACTAGAACAAAACCAACGGTCTGTTTTTCTTCCCCTCAAATATCTATATATTACCAATAATGAAAAATTCCACATTATTTAAATACTAAATTATCAATATATGGTAATGCAAATATTTGCTAAATTTAAATACT

GAAATAATTCAGAAGTGAAATAATCTAGTCCTCATTATACCCTCAAAACTATGATGAATTTATATTTATCATTTTCCCCTAATTTTTATATATAGGTAAAATCTGATAAAAGTAAACTGAGTTTCTTTCACAGTATTTGCTTATTCTAAACATTATCTTCAACTTTGATTTATGGTATCAGTTCAAATAAAGTAAAATCTCACATTTTAATGTATTAATTCACCATTTCAAGCTATGATTTATATTTAAGATTTGAAATAAATGGAGCCTATTATAAAAATTTTCTTTGAGGTGTGAGTCAGTTTTCTTCCCTCTTAATGCCCTTTCCCTTTTCCTCTTGGCACTGATAATTCATCCCTTAATGTCTCACTGTTTTCTCTGAAAAGTTCCCTTTCTCTTCTTATAAAAAGTAAGTATTCCATGGGATTTTTACCAGACTACTTTTAAGACATACCACTAGCCCTGATTTTGTTACTGTCAGGCTTTGTGCTGTAAGTGTGACAAATATTTC

>ENSCHIT00000004774 gene=ENSCHIG00000003577

CGGAGTCCGCCACCCCCAACCCCCACAGCCCCAGCGCCTCCCCGGGCCCCTCCCCAGAGACTTTGATTTCATCCCCTCCCCGCCCCGGGCGGGCTGGCAGGTGCGGGGGTGGGAGCCGTCTCCTGCCGGAGCGGCTTTGCCAAAGGTCCGGCTGCTGACGTCGGAGCGCCCGCTCCTGTCAATGAACGGCAGCGGCAGCCTGGGGACTGGCTGGCAACGAGGCTACAGACGTCATCGCCTCCTGTTGGACGCGAGGGGGGGTGTCCGAGCCGCAGCTCCGGCTTAAAGGAAAACTCTCCAAGTTCCAGGCAGCCGGGGAGCGCCGTTCCCGACAAGCACATGCACCCACA

GCCCGGTGCGCGCGGGCACACGGGCGGGCACACTCGCGCACTCGCACACACGCACACTCAGACACGCACGCACCTTCGCACCCGTGTTCCCTATAGGTGTGCGGTCCAAAGTTGCGGCCGCAAGGAGGATGACCAGCGCGAAATGAGGACTGCCCCAAGCCTTTGGGACGTGCCTGCGGAGCGGAGCCGGAGCGAGTGTTCTTGGAGAGGTCATGAACTCGGGCCACCCGGGCTCATGGGCAACTTGTTCTGCGCAGTTCTCAATGGAGCCCCGGACCCACCCGGCTCCTGGCTCGCCTGCTTCCCACCCCCCCTTCTTTGCTGCTCCTCTTTCTTTTAAATGTGTGAAGTTGAAGTGGAAAGCAGATAGGTGCTTCGCATACTTTGGCGGATACAGTGGTGTCCCGAATCCGTGGGTTCACTTTTTAAGTTGCCAGTTACCCACAGCCAACCTTGATGCAGACACATTAAATGGAAAAGTCCAGAAGTAAGCGATTCATGAGTTTTGACTGGTCCCTTCTGAGCCGCAAGGTGGAATCTGTTCTCTCCAATCCGCTCGGGACACGGTCATCCCTTTGTCCGGCACGTCAGCCCGGTGGGCACTTAGCAGCCCGCAGCTGTCAGACGGGCGCTTGAGGCGTCACGGGGCTGCAGTCAGAGTGACTGACATACTTGCTCATGGCCCCCAGGTGCCAGAGGACTGATGCTCGCCGCTGTTCTGCCACAGAGAAGCCGGACAGTGCATCTTCTGAGTGAAAAAATGAAAGTTCGTCAGTAAGGAAAGAAAAAATACGTGCTGAGGTTGCTAAGATCTACTGCTAAGAATGAATTTTCTATCCGCAATGTTTTAAAGAAGGAAGAAGAAAGCTGTACGGGTTTTGCTGTTGCATCGAAAGCTTTAAAAATTGTGGCCATGGTCCATGATAAGTGCTTAGTTAAGATGGAAAAAGACATTAAATTTGTTATGTAAGATATTTTGAGAGACCACATTCACATAACTTTTAGTATAGCACACTATAATAGTTGTTCTATTTTATTATTAGTTGTTAATCGCTTACTGTACCTAATTTATAAATAGGTATGTACATACAAGAAAAGGGCCGCCAGGTGGCGCTGGTGGTAAAGAACCCGCTTGCCGGTGCAGGAGTAGGTTTGCTCCCTGGGTCGGGAAGGTCCCCTGGAGGAGGGCAGGGCAACCCACTCCAGTATTCTTGCCTGGAGAATCCCATGGACAGAGGAGCCTGGTCCATAGGTTCACAAAGAATTGGACATGACTGAAGTGACTTAGCCTATACGTACAGGAAAAAGCATAGTATATATGGGATCCTGTACTACCGTGGGTTTCAGGGGTCTACTGGGGTCTTGGAATGTACCGCCCTGTGGACAGGGAGGGACAGCTGTACTGATGTAGAAACAGATGCAAGATCAGAGGACGTGGTAAGTTAGTCTTTTTAGTCCCTTTGTCCTCCCCCGCAGCACGCGCACGCACCTGTATATCTGCACACACGGCGTGGGCCAGGGCTGAGGGCACAACAGAACATAGCACTCACCTAGCTCTGCCCTCTGCTGCGGCCACCGGGTGGAGGCGGTGTGTTGGAACAGCCCAGGAAACCTCGTTCAACTGTGACCGTTACCAGCCACCCTGTCCAACCCACAGGTTCTGCCTCCATAATCAGAACTGGTCTCCAGCCCTTCCCAGGGCTTCCAGGCGATCCCAAAAGGCCACCCTTCCCCCAGACTCCGCCATCAAACCCAGCACACACACAGACACACATACAAGGCAGGCACCTGTTCATTTGTGGACCCTGATGCTTAACAACCAGACTCTGATCTTGACCTTTCTTTGCTGATCTTGACCTTGTGTTCACTTACACTTCCAGTGGATTCTTAAAAAGAGATTGACATAAACAATTTGGAATCGTAGTAAAAGTCCAGAAGATCACGCTATACACACCTAAAATTTACAGGCCTGAGGCTGGTGTAGCTTCTTTTGTTTGGTGTACACTGCCATCTAGTGGTGAGATTCTGCATAGACGTGCAGTTTATACCCCGCGCTGCGTAGTCCCACAGAATTGTGTAATTACACCCCACACCGCAGCATATATAAACCTTGTAATTAGGATGCCACTGTCTTCCCTCCCTCTGTGTCTGTATCCTTCCCCTGTGTTCTTTGCTGTATTCTTTCCAATTAATCCTGGAGCCCCCACCGCTGAACATGTTTGCAGTAATTTTCCTAAACAAAGCCTGGCACATTGTCTTGTTCAATTAATCTGATCCTTTGTTTTTCAGTTTTTAAGTTGTGTCCAGCCTTTGCAACTCCTCCGTGGACTGCACCACACCAGACTTCCCTGATCCTCACTGTCTCCCGGAGCTTGCTCAAACTCATGTCCTTCGAGTCAGTGATGCCACCCAACCATCTCATCTTCTGTTGCCCCTTCTCTTTTTGCCGTCAATCTTTCCCAGCATCAGGGTCTTTTCCAGTGAGTTGGCTCTTCACATAAGGTGGCCAAAATATCGGAGCTTCAGCATCAGTCCTTCCAATGAACATTCAGGGTTGATTGTTTTTAAGATTGACTGATTGGATCTCCTTGCTTTCCAAGGGACTCTGAAGAGTCTTCTCCAGCACCATGATTTGAAAGCATCACGTTTTCAGCACTCAGCTTTCTTTATGGTCCAATTCTCACATCCATACATGAGTACTGGAAAAACCATAGCCTTGACTATGTGAACATTTTTAGGCAAAGTGATGTCTCTACTTTTTAATATGCTGTCTCGGTTTGTGATAGCTTTTCTTCCAAGAAGCAAGTGTCTTTTAATTTCATGGTTGAGATCACCATCCACAGTGATTTTGGAGCCCAAGAAAATAAAGTCTGTCACTGTTTCCATTTTTCTCCCATCTATTTGCCATGAAGTGATGGGACCAGATGCCATGATCTTAGTTTTTTGAACGCTGAGTTTTAAGCCGCTTTTTCACTCTCCTCTTTCACCTTCTTCAAAAGGCTCTTTAGTTCCTCTTCATTTTCTGCCATAAGGGTGGTGTCATCTGCATATCTGAGATTATTGATATTTCTCCCCGCAATCGTGATTCTATCTTGTGATTGATCCAGCCCAGCATTTCGCATGATGTACTCTGCATAGAAGTTAAATAAGCAGGGCGACAATATACAGCCTTGATGTACTCCTTTCCCATTTTTGAACCAGATCTGTTATTGTGGTTGTTAGCAGAAGGAGATCAGTTACACTTGATGTGGGTTAATTCCTGTGTTCCTCTTAACTGTGTGAAAGGGCATTTTACATAAATCACTCCAGCAACATTAAGACTAAGTTTATACCACATTTCGCTTTCTTTGTGAGATTCAAACAAACCAAAGTGATTCAAATTGATTGAAAATAGGTGTGTAGTATATTTTCAAAGCAGAAGGTGGGAACTCAAAGGTGCCTGGCTTTTGGCGCGGACTCTGCCGCCCACCAGCGAGCTCCGTGCTGGGCAAATGATTTAGACTCCGTGTCTCCCAAACAGACTGGGAGTGAGTGGCCTCCAGACCCCCGACCACCTCGCTCCTGTGGCCCCCCGAGTGGCGGCTAAGCAGGAGTCAAGCTGAGGAGTCAAAAATGGATGAAAGATGCTCTTTTCAGTCCAAGTTTATGACATTTTTTAAAATTTATTTTTAATTGGAGGGTAGTTGCTTTACAGCAATGTGTTGGTTTCAAGTCCTAGGAAATTTTGAGAGAGAAACATGACTTTTAAAAATATAAATTTCTTTATCTTAATTGGAGGCTAATTACTTTGCAATATTGTGTTGGTTTTGCCATATATCAACATGAATCCACCACGGGTGTACACGTGTTC

CCCATCCTGAACCCCCCTCCCACCTCCCTCCCCGTACCATACCTCTGGGTCATCCCAGTGCTCCAGCCCCAAGCATCCCGCATCCTGCATCGAACCTGGACTGGCGATTCATTTCTTATATGATATTGTACATGTTTCAATGCCATTCTCCCAAACTGTCCCCCCCTCGCCCTCTCCCACAGAGTCCAAAAGACTGTTTTATACATCTGTGTCTCTTTTGCTGTCTCGCATACAGGGTTATTGTTATTGTCTTTCTAAATTCCATATATATGTGTCAGTATACTGTATTGGTGTTTTTCTTTCTGGCTTAATTCACTCTGTATAATCGGCTCCAGTTTCATCCACCTCATTAGAACTGATTCAAATGCATTGTTTTTAATGGCTGAGTAATACTCCATTGTGTATATGTACCACAGCTTTCTTATCCATTCGTCTGCTGATGGACATCTAGGTTGCTTCCATGTCCTGGCTATTATAAACAGTGCTGTGATGAACATTGGGGTACCCGTGTCTCTTTCAATTCTGGTTTCCTTGGTGTGTATGCCCAGTAGTGGGATTGCTGGGTCATAAGGCAGTTCTATTTCCAGTTTTTAAAGGAATCTCCACACTGTTCTCCATTGTGGCTGTACT

AGTTTGCAGTCCCACCAACAGGGTAAGAGGGTTCCCTTCTCTCCACACCCTCTGCAGCATTTATTGCTTGTAGACTGAGAAACACAACTTTTAAATGCACATGCCCAGGTCCGTGTCCCCCAGTCCCCTGAGAGCCCAGAAGACAGAGGCCCTGGTGACGCACACATGGTTGTGGGTTCTGGGGAGGGGTTGTCTCTGGGTGAGGGCCCCACGACCTCTTTCGGCCTCCGGGCGGTGGCCAGGTCAGCGGTCTGCCTTTGCACGCTGCCTTCCTCGCCTCCTCTCTGCAGGCAGGACCGCTGTGTTCGGGGAGTGGGGAATTCAC

>MSTRG.1201.6 gene=MSTRG.1201

GAAGAGGCGGGGCGCCTGGAAACGGCCGCCGTAGAGTTGGCGGACTTGCGAAGGAGCGAAGATGGCGGAGTGAAGCACATTGAGGCTATCTGGCCTTTGGGCCAGGGGGCGGGCCGGCCCCCGGGCGCTGGAGTGCTTCTGCCGGCCCTTTCGAGGTAAGCCGGGAAGGCTAGGGACGGGGAACTAGCAGGAGGTGGCGCGGTCCAAACCATCCGAGGAGCAGAGTGCAGAGCCTGCCTGCAGCTGGTGCGGGCCTTGGCTGGGCGCTGGTTTCCTTCTGAGCTGCCCTAGGGCGGCCGCCCCTGCCTTTGAAGGTGTGTTGCACCACTTCTCAAACCCGTTCCGCCACT

GACCTCAGTGCCTTGGGCTTGGCTGCGTTTGTGCTTTCGGCTCGTTACCCAGTGGTTGCACCCACGCTGTTGTATATCTGGCATTTTGTGTCGTTTATCTTTGAAGAACATCTTAGTTTTATTGGTTTGTTTCTGTTCTTCGTCTCACAACTTCTTGCCCACTGTTTTGCCTTGAGTCTCGGGTTTCAATCGAGGCCTGTCCGAGTTCTGAGTATTTTGTGTGCCCGTCCCGTTGACCTTGAGATTTGATGTAACAGGTCAGGTTCCTCACCCTCCCACACCACCCCGCCCGGGGATTTCAGTTTGAGTCTGTGTATCGTGTATGTACTCTTGCTTAACTCTGTTACATCCATTTATTCCCACTACCACCTCTCAAACTTTTTTAAGGTCTGAGAAAGCAGCTTTGTCCTTGTATACCTGATAGTTGCCTAATGGCGCATTAGTCTCGGCTGACTAGATAAAATTCACCGTAGTTTTCCAGAACTGAAAGAGTCAGTGTACAGTTTAATTTATATATATATATATATAAATATATAAATTTTTTTTTAGAATGAGGAAGAGTTTGGGGAATTATTATTAGAAAGTAATTTGTTTAATGGGGGGAGGTTGCAAAGTATAATACTCAGGCATTTCTATTCTGTTTTTGTTTAACTTTATGGGATGTATCTTTTTGTAATTTGAGTTGTTATGACCCCTCACATTCGGTCTTTCAGTTAAATTGTGGGAAGGGTTCCTGTTACTAGGTTTCTGAGTTGGTGAAGCCTGTTTGAAACTGGCACTTTAGTTAGGCCCGGTTCAAATGACGTGTCGTACTACAAATGAATGTGACTGGTAAACACTTTGTGCTGGCTCTGGATTTAGCAGACCTGAAAATACTGGGTGAAGAAATAAAAGCTTTTTGTTGCTTTTGTCTGGAGTAGTTTTTTCCTTTCATTTAGGAAAATAAAGAAAATATGGTGCAGAACATTCTTCTTCCTACCTTACAAACAGGGGAGTGGTCATCTTTACTAAAGCAGAGGTCTTCTGTGGGAGGAGAGGGCCCACAGCATCATCAGATTCTCAAGGGGAACCTTCTTTAAAAACTTGAGAACCACTGCTTCAGGAGTTTTGACATCTTTATAAAATTAATGCCAGTGCAGATAAAGAGTAACTTCTTTTCTGATTGTTCCTTCCAAATAGTGTTTTATAATGTTTAAGACTTGTTTCATTTGGATAATTATGCACATGGATGCATGTAGCACACATGAGGGCTATGTCCTTACTGTAATTTTTCTTTCTTCCCCTTACTGCAGGAAGAACAATTTCCTTGCCATTTAGGTGCTTTTATATTCATCTCAAAAAGAAACAAAATGCTGAACTCGGGATTTGGCAAGTGAGTAAAAGTAGCTAATGCTTTTAAATACTGTTAAAGTAGCTCAGAAATAGCTAGATGGAAGTGGAACAAGAAATCATTAGCTTCAACTGAGAATGAATATCACAAGTGAGTATAAATTATTTAAGCTAAATCATTTATTTTCAACAAAACCAATGTATTAGATATAAAAGAAAATAAAAATTTGATCATTTTCCCAAACCAATAATTTTTATTGGATAAGGAGGAAAGATTTAG

AGTATATTCACTTAAATTTGAATTATAAAGCTCTTCAGTACACCTTTATTATCTCTTGGTATGAATCTATTCTTCTGAATTTACCACAGAAGTAAAGTACATTCTGCTGCTCAAAGAGCATTAAGTGATAGTGCTTTCATAGTAGACAGTAACCTTATGTGCAAATTAGGGTTCATAGCTACTGTATTAGTTTCCTGGGCTATAAAAAAG

AACTACTAAGTGGGTGACTCGGAGATCAGACATCTAAAATCAAGGTGTTGCGGGGACCATACTCTCCCTGAATGCACTTGGGAAGGATTTGTTCCAGACCTTTCTTCTAGTTTTTGGTAGTTCTTCGGCTTGTGGCAGCATAATTCATCCTTTACAGGGCATTCTCCCTGTGTATGTCAGAGTCCTAAATTCTCCTTTTATAAGGACACTAATCAGATTGGGGCCCATTTGGCTTCAGTATGACTTCATCTTAATTACTTATGCAGTGACCCTGTTTCCAAATAAGGTCACATTTTGAGGTACTTGTAAGACAACATACGAACTTGGGGATGACATATGACATAGTTCAACCCATAATGTCTACTTAGTCTTACACCTTGATTATTGATTATAATATATGAAAACATTGAACGTTCAACTGCTTTACGGATTAAAATCTTGTATCCCAATTGTATAAGGTGCTTAAATATAGTCGAACTCATAGAGCCTGACAGTAGAAGGATGGTTGCCAGGGGCTTCAGGGAGTGGAAATTAAGATTTATTTAATCAGTACAGAGTTTCAGTTTTGTGATACGAAGAGTTATAGAGATGGATGGTGGTGATGGTTGCACAATAATGTGAATGTACTTAATGCCATTGAGCTGTACACTTTAAAGGGTTAAGATGATAAATTGTATGTTATGCATGTTTTACAATTAAAAAATGAAAAAGGTTAGTGAAAAGCTCCAACTATGTATATTTTAATATTAAGAAAAGGAATAAAAACGGAGGAAACAGAGTAAGTGCCCTTTAGTTGTAAGTGTCTTTATCTTATGCTTTTAGAGTAAAGGCTTTGTTGCTTGGATTTATAACCTGAATTAATTAAGTCCATGCAGATTGTTGAATAAAAATTCAGCTGAGTTAATCAATTTTAATTGAAGTATAGTTGATTTACAATGTTGTCTTAATGTCTCCTATACAGTAAAGTGAGTCAATTATACATATACACACACACACACATTCTTTTTTATATACTTTTCCATTATGGTTTATCCCAGGACTTTGAATATAGTTCCTTATGTTAATACAGTAGGACCTTGTTGTCCATCCATTCTGTATATGATAGTTTGCATCTACTAACCCCAAACTCCCACTCCATCTCTCTCCTACCTCAACTCCCCCTTGGCAACCACAAATCTGTTCTCTATGTTTGTGAGTCTATTTCTGCTTCATAATAGGTTCATTTGTGTCATATTTTAGATTTCACACATCAGTGATACCATACAGTACTTCTCTTTCTGACGTCACTTAGGATGGTAATCTCTAGTTGCATCTATGTTGCTGCAGGTGGCATTATTTTGTTCTTCTAGCTGAGGAGCACTCCATTGTATATATGTGCCACATCTTCATTCATTCATCTGTTGTTGGACATTTAGGTTGTTTCCACATGTTGGCTGTTGTGAATAATGCTGCTGTTTAACATAGGGGTGTGTGTGTCTTTTTTAAGTATGTCAGCAGAGTAAAGATCAGATTGGCGTCACTAAATGATTCATGAGTCAGGCAGCCTCTCATCTAGCAGATAGAAAGGACCTCCACTGAGCTGTAGAAGAGGAAAGGTGGGAACGGGGCAGAAACAGGAAATTATTAGCAAAAAATACAGTATTTCAGGGAAGGTCATCCTCCTAAGAGGAACAGAAGGGTTCTGCTGACCGGGCAATTCCAGACTGACTGGTTAAAGGTTACATTTCTGCAGGAGGTTGAAACTGCAGTTAGGTTAGGTATTAAAGTCTTGGTTTGGTGATGTGGGGTTAGCACAAGGGACTCCATATTGGGCCTGAGTAATAGAAATTTTACATTTGTATAGCAACCTATAAATCTATAGGGAACTGATAAGCACTTATCAGTGCTTTTGTACTTAGAAAGTAAGTGCTTTTGTATTTACAAAGTACCTTTAAATTCTGGTACAGAATGATCTCTAGTATACTTTAAAGGAAAAAGCAGGGTGCAGAACAAGGTGTATAGTGTACTTTCATTTATAGGGAGGAAATAAATCTACTTAAGCAGATCTATATTAGTATAGTCTATCCATATTTGGGGGCAGAAACTGTCTAACAAACCCTGGATTTGTTTCAAATAGGACACTTTAAAACTTAATTTGGATGACAGTGAGACCCACTTTGTTTTATCAAATGTGTTCTGGATTTACATAGCAAAGTAAGAAGGTACTTAACTCCTATGGTATATCATTCTTCATGTCCTTAGAGGAAGCAAAGTGTATGAGCTATTTATTGTGCATAACCAATTACCCAAAATTTGGTGGATTAAAACAACAGTAAACATTTATTATCTTTCACAGTTTATGTGGGTCAGCAGTTAGGGAGCAGCTTGGATGGTTTCTGGCTTGACATCTCATAAGGTTATATAGTCAAGATACTAGCTAGGATGGTAGTCATCTGGTCTGATTCTGTGGTGGCTCGTTCACATGGATAACAAGTTCATGCTGGTGGTGGAAAGCCTCAGTTCCTTCCACTGTCAGCCTCTTCCAGGGCTGGTTGAGTGTCCTTATGATACAGCAGCTGGCTTATCCCAGAACAAGGCAGGAGCCTCAATCCCACCTCTGACCTTGCCTCAGAGGTTACTCACTCACTTCCTCATTCTTTTGCTCATTGTTTGAACACTAACTAGTATACTGCAATACAGTTTTGACACTAACCATCTGGAATTAATGCAGACCCCCCCCCACCCAAGTCAAAGGACATGACCCCCAGGAAGACTGCCCTTACTTTAGATGTCAGCTGTATTTTGAAGGATCCTCAGGACA

CTTCTACTTCTGACCAACTGGCTGCAGATTCAGGAATTCCGATGACCCTGTGCTTCTACATTGTCTCCTAGAGTGGGGTAGTCCTGCTTCTCTTATCCAGTCACCTTCCCTGTTGAAATGTGGATGTTTCTTCTCTACACGTTGCCTCCGTGACTTGCACTGGAAAGGCTGCTCTTCTTTTTGCTCCCACTCACATTCAGCACAGAGAAGCCCTGGGCACAGTCCTTCCACCAGGTTCTCCTGGAAGAGAGGAGCAGCTGAATCACAAAGGAATTATTTCAAAAGCTGAAGGCTGATGTCTCGTTTGAAGCAGATGCTTTGATGGTGATATTTGTGAATTCTTTACCTTTTGTTCCAAACAAGAAGACTGAATATAAATAGCAGGTATCGGTATCACATCAGGGTTCTGGGGTTTTTTTGTCTTCCAGTTTTAATCACTATCATGATAAGCACAGTTAAGACTTAAAACAGTAAATTCCAGATATATGTTCTAATTTGAAATGAAAGAGATGCTTAAAGAGATGGCTTACATATGTATCTAAATCCTGGCTTATCCTTTGACATGGATTTACTAGATAAGAACAAAGGTCCCGATTTGCAATAAAACCGTTGATGTGATAAAGAAGTAAAAAGACAAGATATCACAAGGTTACTTCCATATGTGAATAAACCATCACAAACCTATAAATAGCTGTTTTTACATATGTAAGTCCACAGTTTGGGATGATAAGAGGAATTTTTCTCCTGGAGAAGGAAGCTGTGGTTGACTTCATTCTCAGAAATCTTTGGATATTTGGTATTTTTAAATGTTTTTTCATTCATCTAAGGAGTGACCACATTGTGCAGATGCTAACCTTATGAATGCATGCTTGGGTTTAGTGAAAGATTGGTAAAAGGAAACAAGTTTTGACTCCAGTGATGGAACCCTCTTCACCTCTGGAGCATGAGAGACCTACTATATTACAAAGTGTTTCATTCTT

TTTTGTGGAGTCTTATATAGCGCTTGAGACTTGGTTTTACATGCTGCTAAGGCTGGAAGAAAAGTGGCATAAGTGGATTTTCTGTTCAGGGGATGTGATGGACCCTACCGTATGACCTGCCTCAGCTGTGGACATAGAGGAGTGGAGGAAAAAAGGAGGAAACTTTCCGTATTACTCCTTTCCCTTGGAAAAATAAGTGAAAGTGCTTTATGCAGCTCTGAGTTTATGGCATTTCACATTCTTACAGCTCTGTTTTTTGTAAAAGCATAAATAACTGTTTTCCTTTGACCCAAAATGGGTTCTTGGCTATTTGTTTTTCTAGGCAATAGGTGTAATGTCTGACTGAAAAGGCTTGGACTCAGGTAACTTTCTCCAGTTGGAACTGAACTTTCTCATCAGAGAATGGACCTCAGAAGAAATGTACCCAAACGTGATTGGTTCCGTGTAGATTTAGAAAAATTCAGAGTGTGAAAAAAAGGAGGGGAGAAACCCTCAAACAGGAAAAATAATTCCAATAAGAAAAGTAATGCACTTCCTTTCTGTGAAAACATGTATTATCT

GATGAACAGTCTATAGGGTAAAGCTGTCCTTGCTTCCAAGTCAACAGAGGACCACTGTATGAATTTCCAGGTCTTTACATAAGAAGGAAACCTTTTATAGTAAGTCTGTGAGTAGAGAAAGAATTTAAAGCTGCTTCGATGAAATTAAGATCAAATCATCTTAGATTATCTCTGTGATATACAGAATGTTTGCCCAGGGCCATACGTGCTGTGCTGCTGAAATCTTGGGAAAGATTTATCAGAATTTGAGAGTGATAGAAATAGCCAAAATTGGAAAATAATTGAGTATTCTTAATCATTAGTTAAATTGGTCAGCTAGTTTGCATTGACATAAAACATTTTGGCAAAATAATTACATGATATAGATAAAATATAATTTCCCTTAATTGCGAGCAATTACTCAGTGCTGTAAAGCATAGTCAAAGTAGCTTGGATAAAGGTAACTTAAATTTTTCTCTTAGTAACTTCTCAGCCATTTCCTGATGTTTTCTCTCAGTATTTTGGAATTGTTAAAAACTGTAGGAATGCTTTAGGAATGGAACCTGCCTATATAAGTCCCGTTCGTACGTTGAAAATATCTACATTTGTTTGTTAAAGCCATCTCCTCCCCCCGCCCTTTTTTTTTTTTTGGCAAGAATCTTTTGGCAGGGTATAGCAGAGACGTGTACCAGCAGAACCAGTGAAACTGTCTCTTTTCCACTTGTTTAAATATGTGTATCAGACTCATCTTCTCAAGTTGCTTTACATATTTTCATTTCACAGCTAAGAGACCACTGCTTAATGCTTTCTTTCAAATTGTTTCTGCATCTTCCATGATTTTCATCAGATGGCACTTAACAAAATGGGTATATTCCAGTTGTATTTGCCCGGTGTTTACTTACTGCATCCACATTGTTTTCCCTGATGTATTTTGTTCCACTTCATGATGGCAAAAGCTATAATTTTAGGCAGGACTATGCCTCTTTCTGTAGCTGTGCCTACTTCCTTTACTTTGCTTATCCATGTTAGCTGGCATTTTTTTCTTTAAAAAAAGTTGAACAAAAACGGGATATGTGCAATAGAAATATATATATGTATATTTTTTTTTCTAAATACTTGTGGACAAATGTTACAAGTTGTTTAAGAACAACAAAATCACCAATGTCTTCCATTTTGAGATGTGTATAGTTTTGTAAGCATTAGTGCTTGGTAGCATATTGTAGTGCCATGTTAGGGGTTAGTGCATGAACTTAGTAATACTTTAAACTTCAGGATGAATTACAGAAAATAATAGTGTAAAAAGATTGGGAAATCTAGACCTTTTGTGTTTTTTTCATAAATATCTGAATCTGTGATATTCTCCCTGGGGAAAAAGAGATTAAAGCCAAAAGTACTCATTTAGGAATAGAAATGTGGGGTCACAACATGAGATACTGTCTTTGAGGATATTTAAGCTTTTCATGAGGTAGCTGGAAAGCAGTTGCCTTTGTGTCTTTATGAAACCTTACATCAAAACAAGGCCCTATTTCATGATTTGGCTTTGCTCTCTCAAGTTTGAAGTGAAATTGTTCTTACACTGTTTTCACATTGTATGTCTGGTAGGTTTATTGTTTTCCCACTCCTTTTGCATCAGATTATTTTGTATTTTGCATGTTACCAAGATTATGTGTTTATTGCTCAAGTGACTGTTTTTCTGATCTGCAGGCCATTTATGCTTCCATCTACAGTATGCTACGGGTTAACAGAACAACACTGATCAGTAGTTCAGAGTGTCCTAGATGAATTTCTCATGGGTTTTTCCTGTTTTTAAATCTGCTGAATTGTGTATTGATGGGATTCTAGACCTTTTCATGAGAATTGGAAATGAAGCTAAATGGAGCCTACCTAGGCTGCCATGTGTTAACTCTCTGCTTGGTTATAGTGATTGCCTCAGACAGCTTTCACTTTTTTCTGCTTTCCTCATAGAAATAAAAGTTTGTTTTGTTCTGAAAGCATAGCTTTCTGCAGAAAAGAAATTCCTACCTCTAAAAGCTTTCTTGAGAACTTGGAAATGGCAGTTTTCTGCTGTGGTTTTTAGATCTCAGGAGAGTTCAGGATAGACACAAATTCTACAGTTAGTATGATAGGAAATGCAAAACATTTTTAATGTGGTGTGACACGTGCAGAAGCAGGCTTAAACAAATTGACTCTTGGGCAGCAGATCTCCAGGGACCCAGGTTTTAGATTCTAAGATTGTACTTAAAAGTGGTTGTACTCTTGGT

TCAAGTAGTTTAGTGCCTGGGAGAATCCACTTCTGCAAAGCATTTAAACGTTTTTAAAAATACCTCTGCAAAGGTAATGAATGCAAAATAGCAGAGTATATAGGAAAGGATATTTCCTATTGCAAAAAAAAAAAAGCCATAAAACAAGCTCATAATTTTCAGGAGGTAGAATTTCAGTAGCAATAACAGTTTTGCATGTATAATAAGTTGCATTAGCAAGATTGTACAATAGACTACTACTTGAATTTGAGACTGCGATCAACAGAATTCCATGAAAGGAAAAAGATGTTTTAGATAAAGAGTGTATAAAGGTACTTAAAAAAAAACTATGAATGTGCATGAATATATCATTATGTGAGAAATAGCAACTTTTGTGTAAGTCAAAGTTTAAATACTTTACTTGGTTGCTAGAGCAGAGGTTCTTTAGAGCCTCGTTAAATTAACCTTATCTGATGCTTAACCTGGAAACATACCCCTGCTGACGTATCCCCACTGAAGCGCATCGTGTGGGGATTTTGCTCATGACGTTTCTTTTAGCAACAGGCCATTTATAAAATGGCATACATTTTGCTTTGTCACCTCATCTTTCTCCATTAGTTTCCTGGGTACATTTTTTAAAAGAGAGAAAAGTTTGTTGTGTGTATATATTTATGGTTTAAAAAACAAACCAAGAGCTTGGATAACTGGCTTTTCCTCATGCCTATTAGTATAGGGGAGTTAGGATTTCAAGGTGTTAAGCAAAAGGATTGAAAGGAATGGCCAAAGGCAGTACATCATCTAATGTAGATGTGCCAAAGCCTGGGGTTTATGACAGCTTATTTTATCAGGAACCCAGCAAACTAGTTTCTTGATCTGAAGAACAACACAGCTCTCACATCTAGATGTAATTGTGGAACAAGGGCGACTACTTTTACCTCATGTTCATCTTGGCCATTTAGTACCTTGAAGATTCCCCAAAAGAGTGTGTTCCCTTGTTTCCCAGGGGGGAAAGCTGAAAGATAAATAGAGTGGATCTAAGAAGATAGTGCCTCTTGAATACTAAATTCTTAAATGGCAACTCCAAAGGATAGAGAAAAGGCTATATTATTAAATGATTATAGCTCCTCTTTATATGTCCTATGCCTTCAGTTGGTGTTAGAGGCATTGGAATAACCAAGCAATATTCAGACATCTGCACGATCATGAGGACA

CCACTGCATGTTACAGTGATGTCAACAGTTGAGTTACTTAGAACAATTAAAGATTACCCTCAACAGCCACATAACAGTTCTAAATGCTCAAATATGCTCTTGAGTTTTTGTGGTCTCAAAGTATGGTTGATTATTTTCCAATATTGTGATCCCATGTTTACTCTTTATGTACCTGTGATATGCAAAATATGAAGAGACCCTTGGCCTCCAGGAGTTTACATTCTCATGGTGCTGCTGGTACAAGCAAATTGCTTCTCGTTTATTCAGAACATTGCTTGGCTGTTAATTATACCATGTTTGGAAGGATTCCTTGGCCAACAGTTTTCAAAAGCAGAGGGCTATGCTAAACTTCAGGGCATTGATTTTTGAGTTTACATTGTATTAGCTCTGCTGTTCATAAACTCCTTTTCCCCAGGGAGCTGTGCAGGTATGCTCATTAATGTGAATCAGAAACCATTCTGCCTAAGAGCATTTTCACCGTCTACACCACCTATGTATGCTCTTCTATGCCATCACTGTCAGTAAATGGCCAAGAAAACTTAACTGCTCCAGAGGACATCTGTCAGTACTCTTTTCCCAAATTTTTACATTATTCTCTACCCGAAAGGACCAGAATTTGCAATTAAGCCTTTTATAACTGAATAGGGTCCAAATTTTAACCTTTTTGTGTGTGACTTTTGTACCTCTGTTCTGAAGCAGCATTGTGATAGATGTGGAGCAGCTGCCTGCATTTCTAAGCAATCACTTTATCAGCCTATATTTTCAGTGGAAACCGCATGTATCCTTGTAATTTCCCCTAATTCCTCTTAGTCAAAGAGTTTACTCTGCATGTTTATTAAAACTGGGTCTGAATGAAAGTACGAGCAATAGTGGCAGGCCCGTAACTCTTTAGAGTATAATATTCCTAATGCATGGTTGAAAATGTACAGTGTTAATGTGGTTACTAGTAATTATATATATCCATTTATTAGTAATGAAGCAATAGAGACTTAAACCACTATCTTGAATTTATTGTTTGGATCATTAGTTTTCATTTTCTAAGTAAAGACTCTTTCTTCCGTTGAAAATGGGAGCTAGAACAGGGGCAACCACAAGACATTCAGACTGTCAAGAAGAGAGCATAACAGTGCTCACCCTCTAACTTCTTGCCTGGTTCCATTAGTCCCCAAGCATGTTTAAAAACAAAGCACAAACTAAGCATGTTGAATAAAAAGCTGTTGTGGCTCTAATAATCAAGTGCAGAGTTTCAGAAAGGGTATGGAGTGCCAGTGTGGAAGATGTAACCAGGAATGGGATTTGTCTGATAAAGTTTATGTGGCTTAACTTTGGATCTCTTTAAGACTAGAATCTTGAAAGTGAAGAGAGACAATAATGCACGTACTTCAGATGTTCCAGGATTT

TCAGGTAACTGGGCAGCTCTTTGGATGGTACTGCTATTAACCAGCCGTAAGAGTTCCTTCCAGCAAAGGAGCCCTTTACATTTTATGGAGATAGTCCTTAAAATACTCACATCTGTAGTTTGGTTTCTCTGTTGTTCCTGTATTCAAATTTTCAAAGAAAAGTTTTATAGCCAGAGTTTTATAATTTGTTGCTTAAAGTTTTTTATGCCAGGTAAAAAGGAGCAGCTTTTCCCATTTTGAAGAAAAACCTTGTCATTTATTTTCTTGACGTAGAAAATAGACCAGTTTATTGTGATAGCTTACAATTTTTTGAGATGATTTCTGATTTTGAAATAACCTATGAAAGAACATGTGATCATGACAAATGTACAAATGGGATTCATTGTATCTGAAAGAGGCCTAAGTAGAAGAGAATTCAGAATTAGACTGGTTTTCACTAGGCACCATCCAGTTTCACATTCAGTGATTGAAAATTATTTCTACCTGACTTTAATCTTTGCTGTGGGATTTGATTCCAAATACCTTTGGATAACTTTCCAGTAGTTAAAAATTGAATTACTAATAGTCATTTGACTCAGTTCAGGAAGTGAAATGTCATATCCCATATGTAAAGTTTCTCTTTGGTACTTAATGATAACATGTCTGCCAAACAAACCAGTAAAGATTAAAGGACAGAGTTTCTCTCATCTCTGCTTCCATTATTCCACTAGGGCATATTAAAATGTTAATATTAGAAATCCAAACATCACAGATTTCTCCAGGAACTCACAGATTTCTCCAGGAACTCATACTTCTCCAACGTTTAAGTCAGAATAGATATGTTAAGGTTAGAATTTTCAAAAGATTTTGTCTCCTTTGGTGATGGTATGCCACACCATAGATTAAAATGTTTTTCTCCCAGGGATAAGTATTCTGGCAATAAATTATCATCAGTCCTTAAGCAACAGTAATGTTTTCACCCTTACCTTAAAACCACCGATCACTGGTAATTTTAAAAGATTCATATTAGAATAACCTTCACAGAGACTTACTTCAGTAGCTTGTTTTTACTGTTCAGCATCTTCTTCCCATGGTATATAAGATAATGTTAAAAGACTTTCATTGACTGTTTGGAATTTTACAAAAGGCCACTTACTTTCTAATCTATGCATCTTTGGGATTGTAGATTAAATTTTATTCTTTGCTGTAAAAAGGAACTGCTGTAAGAGTTAGAAACTCTGCACCTGTGTACATATATATTTTGGCAATAAAGCAGCATGGGCTGAGAATGCACTGAAATTTTACTGTGTTATTATTTGGAATGGGTAATTGTAATAATGCAGTAAATTATGGAGATTGAAGAAGGAGCCTATTCACAGACCTGCACTGAGGTAGGCTCCAGATGTCAAAGAAAACAAAGGACCTGGGTTTCTTGATATCAGGTTGTAGCAGTTTCTGGTGTAGTTCAAGGAAAAAACAGGTAACCATATGAATAAAGAGGCACCACATTTAATGTATGCCCTTATGCCATCCCCTTTTTTAACCTAAAGTTAGGGCCAACTTTATGCTAGGTCTAGGAGCAAATGTGTAGTTTCTGGTAAGGAAATAAGTGAGCTAAGTTCAGTAATGAT

AAAATTATTGCAGATAATCCATCCTATCTGAAATATATAAAACATTTAAGATGCTTTTATATTTAACTTTAAAAAGTTAAATAACTAAAGAGGTTTTAACACTAAGATACCTTGTAGGTTTAGGGGAAAGGAGTTATTTCAAATTTTAGAAAGTAGATGTTACAAGCTTCCTTGAGATTTGGCTATTAACAAAGAACAGCTGTGTGGGTTTTGTGATGTGACAACTAATGTAGTTTCTTTGATTTCTGAGACTTCATTTAACACAGTGATACATTCTGCTCCCAGGTCCTATGCTAGATGGTGGAGTTAACAAAAATTGAAGTGGTTTCTGCCATTAACTGAGGAGCTTGAAAGTGTACAACTTGGAAACCTCCAATATGCAATTTTTTTGTTCTTCTAAGTACATAGATTTTTTTTTTTTTTAAAATACTTTTTGGGTGCACCACATGCCATGTGGGATCTTAGTTGCCCAACCAGGGGTTGAACCCATGTCCCCCACAGTGGAAGCACAACAGAGTCATAACCACTGGACCACCAGGGAAGTCTTTTAAGTACATGGAATTTTGAAAATACTCTTATTTAACACATATACAATGGTAAATGATATCTTTACAGAGTCTGTTAATATCAGCCAGTCATTGATATTACACTTCCCAGAGGAGCTCTTATACCACTAAGGCTTGTTTAAAAGTCAGTCATTTGTGAGTGTCAATTAATACAACATTTATAAAAGGCATATACCAAAATTGTAAATGAACATGCCCTTTGACTCTGTTCCACTTTAGGAATCACTCCTACAGAAATACCTGGACAGATATGCAAAGACATGTACGAGGGTCCAGTATAGGAATGGTTAAATACTGATGCATTCATGACTGAATCATATGGAAGTGTTAAGTAATTGCTGAGGCCTGGTATAGAAAGCTGTGACATGTGCTATACAAGAAGCAGGTTGCAGAATAATGTGAAAATATTACTGCCTATTATGTCTGTCCACATGAATTAAAGTTATAATAATTAGCATTTATTGAGCATTTACTATTAAGCACTACTCTTAATTAACATTAGATGTATTCACTAACTCATTTAATCCTCACTGTAGTCCTAGTTAGCTTTTAACCCCTATTTTAAAAGAAGAGTTTAATTCTTTAAGTTCATGGCTAATAAGTACCCAAGCCACAATTTGAATCCAAATCATTTGGCTCCCAGATTGCTCTACTGTGCCTCTCAAAAATATAAGGAAGAGTGTACTTTACCCTTTACTTTGACACACATCTACTATATCTGACTTTGATTCTAGAGTAAACATATGATTTGTATTTGGGAAAAAAATTTAAAGTCAACCATCTTGATTAGATGTCAAGTCTAAGCATTTAATTTTCCATTGAAAGTTTACCAAAACAAATCCAAAGTTAAAATAGCTACATAAGATGAGCTTAATTTAGATAAAATATGTTCAGGTAGACATACCATACACAGTACAAACCCCAGGATGAATCACTAGAGACTATTGGGTCTCATTCTTCAGGAATAGAGCTTTTCTGGTACTGTTTGTTGCCAGAGGCTTCCTCCTTACTTAACCAAATTTTGCAAACTGTAAAGCATAAAGC

AAGTCGGAATTTTCTTTCTCAAATATTTTTAAAGGAAAATATCCTTTATGCAGTCAAAAAGGAAAAGAACTTGCTATTAAACCTACTGGGCACGTCTGAACCATGTTACCCTGTCTGCACGCCACCCTTGGCTGTATCATCTGACTACTAGTGCCAGCCCCTCTGGGTCTGCTTGCCACCTCTCACCCATTGCTCACAGGACAACACAATGAGACTCTGAAAAAGAAAATTTGATCCTGTCATAGGGCGTCCTCCTTTAAAATTCTTCCAAGAGCTTTTCATTGTTCTTGGGATAAAAACAAAGATCCTTCAGGAGGCCAATACGGCTTTGCAGCCTCTGGTTTCTGGACACTATTACAACTCCTACGTGCCTTTTTAGGGGCCTCACTGGGCCCTCATATAAGCCGTTCCCTCTATCAGCTTATTAACTCCTACTAATCTTTCAGAAAAGTTATGACCAACCTAGATAGCATATTAAAAAGCAG

>ENSCHIT00000001640 gene=ENSCHIG00000001172

CCTACTGAATAGAAATTGAAACCAAACAAAATAAAACAAGCAAATTCTCCTCCAAGTGTGAGCACCACAGATGAACTTTTTTCTTGTGATCTTCCATTTTGCAGGATGTTGCCACCGACACTGAAGTCTGTCAACTGACTATCGTTTGGAGTCAGCTGAACGGCTTATACCACATACAGGGGAAAAGCATAAATGTGTGTTTGACTTCCATGAAATTAATGATAGATGAGCTACTAAAGAGAGAGCAAGAAGAAAAGCGAGAGCTGAGAAAATAATAATTTAAATATTATGAGCAAGTCTCTTTACTGCTCACGTGACCTACAATAGTGTTGTCCAGGAGGAAGAAAATGAATCCCTGGAGTGTGTACTACACCCGTCTTTATCATCTCAAATCTCATCTCACAGACAGTGAACTCATCTTTGTTGTCAGCATCAGTCATCATGGTGGTCAGCGTTGTTACTATCAGAGCTGGGCAAGAATACCCAGGATGCATGTCAAGCAAGAAGTGGAGAGAAGAAGTGATGTCCCACTTTCTCAGATGGGGCACTTTCTCAGAGAAAGCTTCCAAAGGTTAACAAGTAAGAGTACAATTTTTAAAGATTGCATCCCACTCCGAGAGTTTGGACAGATCACTTGAATATATTGGGCTCCAAGTAGAGATATCCCCTTCCCCCAAGAGAGGCACAGAAAGTGTAAACTCTTTCTTGTGATCAGGCCAATTGCCAAATGTTTGTGAGGGTAGGAAACCCAAGTCATCCAACAATCAAGCAGAGAGTCAATTGTAATAGATACAGTGAAATATGATATGTTATAATTATGACAAGAGGCACTGCAAGATCCCAACCTACGTGAATTATCAGTCTTGCTACTTACCAGAGCACTAATAATACACACATATTAATATAGTTCTTGTTCCAGGGACATGGTAGATTGAGCTAACTAATGCGTAATGTTCTCTTAATGCAGTTATATAGAAATGCTGAATGAAGTAGAATTCAAAACTTTTATATCACAGCAGAGCCCAGAAAAGAAAAAATGCCACATGCCAGGAATGAAGAGTGAACCCCGTCAGACCTGTGCATAGCTGATTGACTGCTTTCCTAAGAGCCTTGATGCAGAGACCCAGGCTCTCTGTGCCTTACAGTTCTATCATCTTTACCATGTAATCTTCAAAGTCACTGTGCTTATTTTCATTAAGCTGATGAAGGATAAGAGCACAGAAGATGGCTTGTTGGAAAGTTTTATGGATCAGACCTACAAACAGCCTATAGTACTTCCGTTAAACTTCTGGTGAACTTTCTTCACAGTGGCTGGGAATGTAGGCTAGATTGTACCAGGAGGAGGAGGAGGAGGAGGAACTTTGTTCGGAGATTATCGTATTGTATAATGCAATACAAGAACCTTATAATGTAATGTTATTGTAAGCTAAGTAACCTTACCTTCTAACATTCTGGATAAATATAAGTTGGAAAAAAAGGCACAAACTCTGAGTCTCAGTTGCTTACATTTTGATGAAGAACAGATTGAGAAACTTTTAAA

TAAAATAATAGCTGAAAGTCTTTGTCTCTAGGTACATTCTCTTTTTACATTATGCTCATAGCTGTAATGGATTCCATCTCTTCCAACAGTTGAAGCTTGATACCCAATTGCATAGCAAAGGAGGATGCTTTACTTTTCCGTTAATGGCTAATAAGTATATTTCTGGATTCAGGAAAACATGTTTTCCTTTCGCTACTCAAAATTGGTGTGTATTTGTTGCCTTCCTTAGAAAGCTATGAGTGCATATAATACAGGAGGTTGAAATATCTGCATGTTTTAGAGATAACTGAGTTCAAGTGCCAAGGCCAAATTATAGATGTATTCAATCAGATGAAAACATTAGTTTTTGTATTTCTGAGCATATTTGCTTTGTAGTCATTCAAGTAAGGGAAAAGGAAATGGCAACCCACTCCAGTATTCTTGCCTGGAGAATCCCAGGGCCGGGGGAGCCTGGTGGGCTGCCGTCTATGGGGTCGCACAGAGTTGGACACGAGTGAAATGACTTAGCAGCAGCAGCAGCAGTATTCAGATAAGAATTTAGTTTTGCACATAGCTTGATTTCTTTGTTTGCTCTCACAGCCAAACCCGACTCCACTTACTAGTCTCTGCTGCAGCTTGCTGTAAGTGGCTACCTGTGTAGTATTACAGGTATTCCAAGTACAGCTTGGTGGAATCCGTAAGAGGATGGGTAAGTATTGTCTTTGGCCTGCCACAGGCAGGTGGTTTAAGAGCTAGCGCATTTCAGTCCTCTTTAATTGTGCCTAGATTTGTTATTAGAGTGGGACAAAGCCTAGGAAACTAGAAATCATATTGGAAAGGTTTGAGATACCGAGGTAAAGCTGGGGGAGGGGTCAGCATCTTACAAGTGTTAGTGATATACATGGTCTGAATCTTCGTACTCTTCTCTTTGTACATTGGGAGTAAAGCCATTAGGTGGGGCTGCAGGGTGGCTCAGATCTGAGAGCAAACACTGACCCCTCGTTCTGCACTCTTGGCTCTCACTCAACCATCTGAGGAGACTTTGGGATCCCAGGCAAGGGAACGTCTCAGAGTCTTTCATGATTCCATTCCTGTCTTTGTCAGAAACTTCACAGAGGATTTTTCTCAAGACCACAGCTGCAGAGGGGTTGTTTCAGGTCAGTGAGAAAGAGGGATCCACTCCTTGGAGTGAGACTAAATAAAACTGTAAAGTCAAAAAGGTTGCAGTTTTCACAGCAATCGCTGAGAATACTCAGGGCATACAGGGCCACTGAACCACTGTCAGCAACATGCGGTGTGGACCTGACCTGGGATTCCCTTACAGCTGGAGCAAAGAGCAAAAGACCATGAAGAGCTGTCCTTTCTGACATCTACAAGTGGGTGTCTGTGATTCTTCCCTTTGTTTCTATTTAAATTTTCATCATTTGGCCTTTATGAATGGACAGATGACTCTGATCCTTGCTTTTAACTTTTTCTGGAGGAACGGGTGAATAGATTGCACGTCTTTTCTGCTAAACCTTGAGACAGGTTTAAAACTAACCTTGTGAGAAGAGAACTAGCAGAGCCCTCAGCCAGTGTGAATCCTCCACTTGGCATCTGGCCTGGAAACTTCTCTCAACCACACTCTCAGTGCTGTCAATCCGCAGGCCTTTCCTCCTGAGTTCCGCC

>MSTRG.3965.1 gene=MSTRG.3965

ACTCTGAAAGAGTATTTGAACGTGACAGGTTAAGCTATTTCTCCAGGGCCCTTTTCTCCCAGGAATGTTGCACCTGGCATTTCAGTTTCTGGAGGTTTTTTTTCTAAGTGTTGTAAGGCATGAGCAGAAGTCTCACCCAGGTGTTCCTGCAGCTTTGACAACTAGTACTCAACTGCCAGCGCTACTTCTCTGAGCATCTCTTGGAGATCTTGCCTA

>ENSCHIT00000009835 gene=ENSCHIG00000007243

TGCATTTCTATTACCCTGGATAGTCTAGATGGCACTTTTTAGATCTCACGTTTTGTTTGGCTTCATCTGGGCCAGTTAGTTTTGTTCTCACCCCTACTTACAAACTGTCAAAACACGCCATCCATTTCATAACTGTGTTCTATATAAAAAAAGAACAACTAGACCATTTTCATCTCAGTTGCTGGGGAAGAATTCCAGTGGAATGGACCTAACAACACAAGGGAACTGGAAGGGAAGCTTCAGGCAGGCTGACTCAGATACCCAGGCTCCTTTCTGCAGTATCTCTGGAGACCAGCAGACGGACAGCAGTCGGAAGGCATGTTGCAGCTCACATTGTAACTCGCTGGAAGAGGGTGATGGCAAAGCAAATGGAAGTCAAAGGGCACAGAACTCAAATCTGCAGCTTCCTGGAAAATAAATTACTTCTAGCCTAGAAGGGTGAAGTTGTATGATCAGTCTTGAATCCTGGTACATGGTGATTGAAGACCTAGATGGAATGTGCTGGACACAGCCACCACCTATGATGCCTTTTGGCATAAATGTTAACCACTCTTTTACAGTTGAGCTAAAGTCCCACTTATTCTACAAAGAAGTTCCTGATTGTCCTTAGTGAAGGGGGATCCTTGGAGCTCCAAGGATCCTATAGCAGAGTTTATCCTCTGGCCTCTACACAAAGCACATCTCTGATAAGTTATTGGTTACGGCATCTTTTCCACTAACAAATGTAGACATGAGAGAGATTCTAAGTTTACAGATCATCGCAGTAAAGT

GAGTCCTACAGGTTGTTCTGGTTTCCCAGGGCACAGAAAAGTCATGTTTACACCATACTGTAATCTATTAAGCATGCAATAGCATTAGGTCTTAAAAAATAATGCATATACGTTAATTAAAAACACTTGATTGCTAAAAAAATGCTAAGTGTCATCTGAGACTTCAGTTAGTTGAAATCTTTTTGGCAATAGAAGCATTGAAAATCACTGATCACAGATCACCATAATGTATTAATAATAATGAAAAAGTTTGAATTTTGTGAGAATTACTAAAATGTGACACAGATACATAAAGTGAGCAAATGTTGTTATAAAAATAGTGCCAGTAGACTTGTTTGATGCAGGGTTGGTCACAAACCTTCAGTATGTAAAAAACCTAATATCTGTGAAGTCAATAAAACAAGATATGCTTGTGTGCATTTAGAATCCAATGAAATTTTCATCTTCGCAAAGGTAGGGGAGGGACCCTATAAATTTAGTACCTGTAACAGGCCCAGTGGCAATCCTAGATACAACAAAGATTAATGCCTATTTGCTG

>ENSCHIT00000005498 gene=ENSCHIG00000004135

CAAGTCCGCGACGGTTTGCCGGCTACATAGAGCACGGCCATGGAGACCGAGAGGAGCTCCCTTGTGGCCCGGGACTTGGAGACCGCAAAGGACATGCAGCTGCGGGTGACCCCGTCGGAAATGAAATTTCTGGACGCGCTGGCAGGGAGGGTTTACCGCCTCCCACTTACCGTACACAATCTCGGCCGAAGCATCCAGAAAATCCGTTTTCAGGAGCCTGTCAAGCCACAGTTCAAACTGATTTTGACCAATCTGGATAAATCACTTGCTTCTGGCCTTCAGACAACAGCTATGGTGGAATATCATCCTGATAAGAATGAAGACGTGTGTGACCAACTTCTTATTTCGATAGGAAATAAAACAATAGAGATCCCTCTAATTGGGTTGATTCCATCATGTCAATTGGCAATTGAGCCAGAAGTTAATTTTGGGACATTGGTTGCCAGTAGTAAAGTGTATTGTAAAGAGATTAGTATCATTAACCATGGCAGTGTGCCAGGTATCTTTAAAATAGAATACCAGGGCCAACTACCCATTGTCATTTTTCCAGCCAGTGGTGTTGTGCAGCCCAAGTCATCAGTGGTTATTAAAGTGGATTTCTGTGCAGACCAGCCCATGGTTGTAAATGAATTGGCAAAAGTGAGTTTGCAAGATCGTCCAGACATATTCTTGAATATCAGAGCTCATGTGGTTGAGCAGATTATTGAATTGTTAAACATAAATAATGATAAAAAGTTGGAATGCATAAGCTTTGGTTCTGTTTTCTTTGGAACGTCAAAGATTGAACGTGCACTTCTGTACAATAATAGCCCAGAACCTGTAAACTGGGTAGCCATAATGCAAGATGATTCCATTGGAGAAGAATTGGGTACAAATATTAAACAAAGAACAGATGTTGCTTTAAATAATCTCACCTACTTAAGGAAAATAAAGAGCATAGATATTACCACAATTATCTCCTGTGTTCCTAATGAAGGGAGGTTACTACCTTATCAAAAGAGTTTAATTACATTTTGTTTCAGCCCAAAGCTAATCATTGATGGTCAAAAGAATGATCCTTCACATAGGCAAGACTATGCTGTTTATTTGAGATTTGAGTCTGTAGGAAGTAAAGATGGGTTTTTGAGAGATGATAATCAAATCATCAAAAGTGACTCATTTCACAAAGTGGAATTAGCCCTGACAGGCTCAGGACTTCCAGTCTTACTACAGTTTGATCCTGGAAGGATTCTTAATTTTGCACCTTGTTTCATGGGTGGACATTCAGAGATTCATTGTGTTATGCAAAATCGATCCAAATCCCTTCCTCTGATGTACCACTTTAAAAAAATTGCACATTTTAAAATTGATCCTCAAAAGGGCAAGATTGATGAAGGATGTATGCAGAATGTGACATGTTCATTCATTCCACATCAAGTTGGAGTCTTTAAAGTGAAGCAATTTATAGAGATTATTGGCTCAGTGGCAGATGAAAATTTGCAGTCTTCATCACTGAAGCCTTTCCATTATATATATTTATATTTTAATAGTGTCTGCAAGCCTTCTACCAAGAAAGTTGTGATGAAAGTTAATCCTGGTATATCCCCTTTGGTCAGTAATCCTACGGGACAGTTTGTGGTGAAAGACTTGGTGCAGTACAAGGACCATGCACCTGTTGCGATGCTTCAGTCAACCATGACACACATTCACAATCATAAGATAAATAAAGAGTTAATAAAGGGTGCACTGATAGCCTTTCCCAATGACCGAG

CTGCAAGTATCCGGTCTGGAGAAGACCATGAAAATTTCAGGACGATTTTCACAAAGATTCCAAGATATAACTATGTGGATCCTGATTTTGCATATACTAAATTTGAAAAACTAGTAAAGAAATCTCATGAAAACTATTATGCAGGATACATTAAATTTTTAAGAAATGTGCGCCTGCAGAAAGAAGCACACAGGAAGCACACATACTCATATAATGACATAGACATAGGATTACATCCTGCATCTGGTCTAAAATCACCCACTGTCTCAGAAGTGGAAAGGGAAGAGGAGTTACCTTCAACACAGTGTTGGATCAAACACAACCAATTGCTAAGAACCAGAAATATAGCATTCAAGGAGACAAAGTCTCTGAGAAGAAAGGTTCTTAAAGGACTTAAATCAGACCCATCCACACCCCATGAAAAACATGATTGCAGCTTAACTTTGACACCCAAGCAAACTTATCAAGTAATTGTTGGGCCCTCTGTCCTTAACTTTGGTGATGTTTGTGTGAACTCAACAAATACTCATCTACTACATGTTATTAATATGCTACCAATTCACATTCTGATCCAATTAAATGTCAATTTGGAAGAACTTCAGAAAACCAGCCAATTTTCATATGTGATTCCACCTACAACTAGTACTTATATTTCAATGATATTTGAATCTCCCACCATTGGAAAATTTTGGAAGTCTTTCACCTTCACAGTGAACAATATGCCTGGTGGACACATCCTAGTGATGGCAGTTGTCCTGCCAGTAAGACTTCAGCTATCTTCTAATGAGCTGATACTGAGACCACAAGGCTTCTTGGTGAAAACATGTTTTAGGGGAACGGTTAGGATGTATAATCATCAGAATTACTTTGTCAAATTTGAATGGCAATCGATAAATACAGGAAAGGAGATGGCATTTTCCATTCGTCCAGCTAAAGGCACTGTTGAACCATACTCCTCATTGGAATGTGAAGTAACATGGCAGCCAGCTTTTGATTCTCCAGAAAGGGGAGAATTTATTCTTCATGTCAGTGAAGGAAACACGCTGAAACTGAAATGTCTTGCACATCTTGGACACTCCAAGGTAACATTTTTAGAGCCACGGATACTCTTTAGCAATATTCCTCAAGGGCTAACAACTTGGAGGAAAGCCATTCTTCACAATGTAGGACAAAACCATGCTTATTTCAAGGTTTGTGACCAGAGTCTTTTGTCTATGATTAATATTGTTCCATCACAAGGAATCATTCCACTGGGGGGACTAACTGTTCTCAATATCTCCTGTACTCCTACCGTTAAAGAAAAATTTGACACAAGAGCAAAGGTTGCTATTCGCTGTGCAAATGACATAGACCTTAGGATTGGTGGATCTGTTGAAGTTGCTGATGTTGAAATCCATCCGAATACATTTAACTTCACTGGCACCTATGTTGGTTCTACTCAGATTATGCCATTTTTACTAAAAAACAAAGGTATAACACGTGCCAGAGTAGAGTTTAATCTGGAAGATGCTGATAGTTTTTCACTGGATTTTAAAGGCAAATCAGGGGAATTCACAGACCCTGCATTTCCTGACATATTTTTCTTGGAGTTAGAGAAACAGACATCTATGGAGTGTGGCATTGCATTTTCTCCCAAAGAAGTGGCAGCGTACAACTTTAGCATTCAAGTTCGAGTTAATTTCTTTGCAGCTTCAGAACTTGACTCTCAATGTTGTTTGTCAGACTCTCATGTGATTCCAAATAGACCACC

AGTTATTCGATCATGTCATATTCAAGCTACTGTGTTGCAAGCACCACTGACATTATCCAGCACTCATTTTATCTTTGAAATCCCATTACATAGTCTGGATCCTAATAACAGGGTCACAAAAACTCAGAATCTGGTCTTACATAATACTTCAAGAAAAGATGTGGAGTGGAATTTGGATATTAGAAATACTGGCAAACTTTTCAAAGATGGTATATTCAAATTTAGCACACTCAGTGGAACTCTGCAACCAAATGAAGAATGCAATGTTGCTGTAAATTTCTGTCCAAAACAGCCTAGAGAATACACAGCTGATATCCCTATTCATTTAAACGATAATCCAGTTTGCTATCGAATGTTACATCTGGTTGGAGAGATAAAATCACCCAAGTTATTATTTGTTCCTCCTTTCGTATTTTTCACTCCTGTTCCTTTGGATGTACCAGCTGTGATGGATGTCAACATTTTACCTCAGAACTATTTCAGGTAAACACCTAATGTGAATTCACCACATCTTCTTCAAGTACTTTTTTGTTTCTGTATGTGTTATACGAGTCATGAGCAATGTCTGACTG

>MSTRG.14879.1 gene=MSTRG.14879

TAAAAGCAAAAAGAGATAAAAAAAAGAAAAAAGCTTACAGATTTAGATGTCTGCTACAGTAACTCCCTTGGATTTACTAAAATGTGTACATCAATAGGTACACGGAGTCCTAAGAAAAAGAGTGCCACTTAAAAATCAAAATTGGACAGTAATTAATTTTAAAAACAAAAGGCTGTCTACTACTCCAAATGAATATAAAATGTCAGAGACCTTATACACTAAGAACTGAAGCCTTGACCAGAGTTTCATTACTCCTGCCCTTGAGCTGCCTTTTCAAGGAAGTATACATTGACCAAATGGCGATTGAGGTGCTTGCTGT

>ENSCHIT00000003656 gene=ENSCHIG00000002650

GTTAAAGTCATCAGTTAATCTTTTTCTGTTCATGACAGAGTTCCTTAGTGACCAGTATCTGTATCTATGATTACATTTCTATGTTATAAATGGTCAATTAGAGATTTTTCAACCTGTAAATAAAGACAGCATAATCAAGAGGAAAGAGACCAGCATTCTAGACTTTCATGTTTACCTAATCTCTTCCTCAGTTGACTGTCAGGAATAGCATCAAAAGCAATTGTGAGCACCATTCTTAAGGCACACTAGTAGTTATTTTAGTAATATATCCTTTTTATTTTGCTTTCCTTATTTCAGTCTCTCTTTTTGAATATCTTATTGTACACCAGTTTTTCTTCTCCCCTTTCTGTGCCTTGGGTTTGTTACTAGTTTCTGCTGACTTGATGAAGTATTGAATAATACTCCATTCTACTTTCCCAG

TACTGAACCACTTAAGATATATACAGAGAAGCAAGACAGATGGAGATTAAAAAGCTAGATTGAAAACTAGGGCTTAGATCAACAACCAATCAAGCTTCCTAATACTTCTCACCGTACAGACCTTGGAATGACCCACTTACCTGGAATCACTGCTTCAAGTGTCGAGCCTGGAGAGTAGGAACAGAGACAAGTAGTAGCCTCCAGCCATGCTTTTAGAAGGCAGTTTCAAGTCAGTGGTAGCAAAGAAAACTAAAGATCATCATAGATACAAGTTGAGATTCGCTTTGTATAATGAATAACTGCGGATTCACCAAGCAAAAATACTAGAGTGGGTTGCCATGCCCTCCTCCAGAGGATCTTCCCAACCCAGAGATCGAACCCAGGTCTCCCACATTGCAGGTGCCTGTGGGAGGTGAGGTGGAGGGTATGAACATCCTGGGCCTGGTGGTGTTTGCCATCATCTTTGGTGTGGCCCTATGGAAGCTGGGTCCCGAGAGAGCACTGCCCATCTGCTTCTTCAGCTCCTTCAGTGATGCACCCTGCTGCTGGTCTCCTGGATCATGTGACTCGGAGCAAAATGATGCCAGTCAGCTGAGCCAAGTGTGTGCATTCCTCCCTCAACCCCAGACAGCTTAATAATGGTTCATTAAGCAGTTAATTAGACTCGCAGTACTTTCAGCCAGGCTCACAGACCCCGCCCAGTCGACACACCTTTGCCATGCTCCTTTCTCATGGTGCTTCTCACTTGGTTTTGCTGTTTAGAAGTGGCCCAAGAGGGATTTTTATATTCTAGGAAAAACAAGCAAACAACTTTTGTATTTAAAGCATTCTGTCGGTGCAGTGATTTTTGGTTTGTTTTGTTGACTATGCAGCAAAAGATGTGCACTAGGGAGCTCAGTGATTCCATGACCTAAATCTAAAAGAAGAGACTGAAAATAGCTGGGCAGGTGCCAGAAACCTACACAGGCAGCACATGTGCAGGACCATAACTAGTGTGGTTCTTTCATGAAGAACATCTTGCTCTCATGACCTGAAGGAAATCCCAGAGACAGATTCAGATGATCGGGTATTGCTTGTGGAGAATCCTTGCACTTAGAATTTTGTCCTGGTCCTGCCTTGGCAAAGTGCCAGTTGGGGAACCTCCAGGGTCTGCAAACTGGTCACTTAAATAGAAATTTTTGTTCTTGTTTTGAGAATTGTCATAATTGAATTTGCTATTTTCTAAATATAGTATTTTATGTATTTCAAGCAAACATTTTATTTATTTACCAATCATCAATCATAATAAAAAAAAACAAAACGGACCTCAGAACTGTGGGCAGGATTGCCGTTCATAAGTGCTGACATCTAGTGGTTGCACTTTAATAAAGTTCTTTATTAAAG

>MSTRG.9526.6 gene=MSTRG.9526

GCCGGGGCGCCCTCCGTCTCCTCTTCCAGCTCCAGGAATAGCTCTGGTTCTATGGGATTCTCTTCAGCTATAATAACTCCCAGGGTCAAGGGTGAACTTTTCCCAGGCCTCAGGGAATAAGAACTGTTTCCTGAGTTACCAACTTCCCTTCTTTCTTCACAGTTGCACTTGGCAATCAATTTGTTAAATTTAAAAGGAGTGAATTGTTATTTTAAAATAATGATTGTTTTGTTTCTTCTTGTTTTGGAGGGTGTACGCATACATCTCTCAGCTGTTAACAGACAAGAAAGTCTTGTGGATATTTGTTCTTAAACACTTTAGTTCAGATTCTCATTTTTATAGATGTAGAAAGCAAGGTATAATAAATTAAGAAAAGAATTAATAGTGTGTAACAAGGAAATGGTCTGTGCAAGGAAGCAAGTTATAATAAATTAAGAAAAGAATTAATAGTGTGTAATGAGAGAAGTGGGCTGTACAAGGGTTCTGACCTTATAAAAGCCAATTTGGTCACCTTCAGCTTTCAGGTTATTTTGGTAGATAATCAGTCCCTGAAAAACTTGAGATGACTACAGGGTTAGCATTTTCCCTACAACCATTGTCCAGGTTGTAATGAAAGTGACACCAAATCGTACCACTCATATGGAGGTTTATTAAGCAGACATTAGCAGGTAGGCATATATTCTACCTGCTAGAATAAGACAACTGTGCCATACATTCTATAGGCTCATGAAGTCCCAGTTCACTGCCATTTCAGATGCTCCACTACTACCTTGCATAAAAGGTTTATTGAAGACGTGACAGCAGAGGCAGAATTTTGGTTACTTTCAGGTCAAAAAAAAAAAAAAAAAAAGGCAAGATGTAAGTCCATTTAGGAAACAAATCTTGAGCATTGTCACTAAAATTCTGGTCCCATACCTTTTTGATTCTTATGCCTCTCACGCCAGAATGAGGAGTGTTTCTATATATGTTAGGCCACGTATGTTTGATAACTACTTTTCACTGATAATCCACTTGAACATTAACTGTTATGCAAATTAGCAAGACATCAGGTGTTGATCAAGAAATGCTCAGTGGGAAGTTTTGTGATAGTTGCCTTGGAAAGCAGCCTTTCTGATTAAAAAAGCCACCTAATGTGGTAAATGCGGTGGTGGTGGGGGCACAATAAGACAATTTTTGAATGGAATGAATTCTTGGTTTCCATCATATGCTTTTTCAGGGTTGCCTTTCAGTGTCTATAACTGTTCCTTTCTTTTGGCTTTTGCTGACTCCTTTTGGTTTCCCTGTTCTAAGTTGTGGCTGAGTGTCATGTTTTATTCTTCAGACAAGGGGGCCTTAATTGTCTGTATCTTCTCCTGACCCTCACTCTAATCCTGTTAAACTAAATGATGCTCTGCTATCATCTCTATGTCAGCAGTATGTTTACATAAACCACACCCATGGTATTTCTGTATTCATGGGGTCATTTCAAGGCAGATGGTGGAATGGGAAAAATAAATACCTATGTTTATACCACTCTCTTGAAAGTCGTCTTCCTTTTTCTTTCTCAAGAAGACATGCAAATCATGTTAATGCTATTTTAAAAGTACATCCTGAATACATTTCTC

>ENSCHIT00000008408 gene=ENSCHIG00000006309

GGGCATCCTAGACCTTCATTGAGGTTTCCAAGGAGATATGTCCTCCTTTGTAAAATGTGGTTAGTAAGGATACTGATTATCTTGAAGTTGCTGTGAAGATTAAGTGCCATGAAGCATTATAGTAGATGCTTAGTAAACACTGGGCTTCCCTGGTGGCTGAGATGGTAAAAGAATCCGCCTGCAATGCAGGATACCCCAGTTTGATCCCCGGATTGGGAAGATCCAATGGAGAAGTGAATGGCAACCCACAATACTCCAGTATTCTTGACTGGAGAACTCCATGGACAGAGAAGCCTGGCAGGCTATAGTCCACGGGATCACAGAGTAGAATGTGACTGAGCGACTTAGCACACACAGAGTTAAATGTTAGAGCCACGGAAATAAATGAAGATGATGATGATGCTGACAGCACCACTGTTACTAGCAGATGTCGTGGTCATAACGTTCCTTTTTTACAGCAGCTAGTGGTCATTTCACGTGTGTCGTTTTCATTTTGCTCATTTCTTTCCAAAAGAGGCTTCCCCGTGGCTGCCCAGCACACTGGCTGCCCAAGATTCAGATCAAAGCTACTCTCAACCCCTGCTGTGAGGACTTGGAAGCACTCTGGCAGGAGGAGGCACTGGGCCTGCCCCATCTCTCCCATCCCCTGCTGGAGCTCTCTTCACCACAGCTCACTCGGGCATCTGCTTTTGACCTCGGTGCCTTCCCTGCGCGGCGGTGCCTGGTGTCTGGAGGAACGCCAGCTCTTCCTGTCTGCCAAGCTGCCTCTCTTCCATCACACAAATGACCTGGGAAACCCCACCTCCCCTCCAGCAGGAGCTCAGTCTCCCTGGACTGATCACAGGAAAGGTCTCAGGAGACTGGTTCAAGAGGAAGACGGCCTAGAGCTTCTAGATGGAAATGCAACGTCCCAGCACTTCTGGCAGTGAGATTAGCAGCACACAGACCTGGATATCTGAAAGAGGCAAGAGTTCTCTTCCCTAAAGACTTTCCAGTCCAGGGACTTCCCTGTGAGTCCAATGGTTAAGAATGCACGTTTCCACTGCACAGGACATGATTTCAATCTTTGGTCAGGGAACTAAGATCCTACATACTAAGTGGCATGGCCAAAAAATAAAGA

CTCTTCAGTCCAAAGAAGACCTGTGGTTATGGTGGTGGAGATGGACAGAAGACCTGTGGTGGGGCTGCTGATACACAGTGGGGTCCAACCAAGGCAGCTCCTAGAGAGCAACCAACATGAGGAGGGAGACAGATTCCTGACTCACTCCCTGAGCTGGCTTGCAGGCTGTAGAGATCTGTTACCTAAGGGAATGGATTCATGAAAAAGGACAACCGCCCCTTAAAATTCACACTTTATTTACATTTATAGAACAGCTTTAAGCTCACGAAGTGCTGTCTTCATATTCTTCTTGGATTCTTAGAACAAAAAGGCAGGGTTAGGTTTTTACCATTGAGCACACTGCCCCAGAGAAGTGAAGTGACGTATTCAAAGTCAACCAGCTAGCTAGCAACAGTGCCAAGGCCAGAAGTCAGGTCTCCTGAAACTTACCCGGAAGTCCGTTTCCCTGACACCACTGGGA

>MSTRG.15892.4 gene=MSTRG.15892

ATGGAGGTGATTGGTAGGTTCTGGCATCTTGGGGAGAGATTCAGTGGGACTGTCATCCTAGGCCATGGGGTGCCGGTTCCCCAGGTCCTGGTGGGTGGAGACAGGCCAGCTTTTCTTCCTTCCTGGTTCCCAGAGGTAGGCGATGGCCCAGACACACCCCAGGAGCCAGGAGGCGTGGGGAGCCCTGACCTGGCGTCCAGATTTACTGGGTGTTGCCCGGGTGTGTGTTCTGAGACCCAAGGGCCCTCGTGGGTGTGAAGAAACCAGAAGCTGAAGTAGGCCTGCCGCCCTCACATCCCACCCAGCCCTGGCTCCCGGCCAGCAACTGCCAGAGTGGTGACTCAGAGCGGCAGAGCCCAGGAGAGAAACAGGGTGCAGGGTGGGGTGTCCCCCTCCCTCATCCAGCCTTCCCAGCACCCCCATTAAGACCAGGACCCTTGAGGCTGCATTTCACACTCCCCTGCACACCACCACCATCTTCCCTCCCTGCTCCCCACCGGTGGGATTTTTCTTTTTCTTTTCCTTGGCACTAGGCTATGGCAGGAAAACAGAAATGTGTGTGGGGCCTGGGATATCTCTACGGTTGGAATATATTGAGGCCAGGATGAGAGAGTGTGTGGTGGGGCACAGTCGCAGGCACTGGCCTGGTGGCAGTGAGGAGTGAGCCAGTGTGGAGAGGGAGCCCCCTCCCATAGAGCTCCCAGGTTTCCCAGGGGCAGAAGCAGGGAGGACCAGGGAGGTCCTAGAAGTGGAGGCGTGTCCTGCACAGCAGCATGCTGGATCATCTGCAGTTGAAAACGCAGAGGCCCAGACAGGTTAAGCGGTTTTCCTGAGGCTGCTCAGGAAAGAAGAGAAGGCACCACCAGGATTTGCCTGCCGGTCTGGCTGGCTGCAAAGCAGGAGTGTGCTCTTGCCATATTCGTGCGCACGTGTGTTCATCGACGCAGCTTTCTGTACAGCCTAGTCTGTTCTCAGTCAGACGTGAGATGTGCAGGAAAGCAAGAGGTGAACTGGGCCTGAGTCCCCAAACCACAACCCGGCTCAGAGCGGGGAGTGGCAGGGTTGGGGATGGAGAGGGGATGGGGAGAACATCCGCTCCTCTTCTCTGCACAGCCCTCAGGCTCAGAATGGTTTTGATACTTTTTTCTGCTTTTCTTTCTTTTTTGTTTTTGGTTGTAGGGCATAGCCTGGGGCATTGTTCCATTGAAGTTCCCTCACCAGGAATCAAATCTTTGAGCTCCCTGCAACGGAAGCTCTGAGTCTTAACCACTGGAGCACCGAGGAAATCTAGTTTTGATACTTTTTAAGCAGTTGAAAACAAAAGATTGTTCCTTGAAACATAAAATCATATGAATGATGATTTCAACCCTCGTAAATTACATGTGTACAGTCGTGCCCATTCCTGTCTGGCTGCTTTGCCGCTGTTGTTCAGTCCTCGATTGTGTCCCCGTGGACTGCAGCATGCCAGGC

TTCCCTGTCCTTCGCTCTATCCTGGACTTTGTTCAGACTCATGTGCCATTTTTTAAAAATTGAGTTAAAATACACTTGACAAAAATTTACCATCTTAGCTATTTTTTAATCATACAGATCAGTGGTATTAAATACATTTATATTGCTCTGCAACCATTATATCATCTATCCCCACAACTCTTTTTTTTTTTTTTCCCGCCCACTTCCCCCCTACTTTTTGGCTGTTCTGTACAGTATGCAGGATCTTAGTTCCCTGACCAGGGTTTGAACTACCCTCTCCAGTGGCAGCCCGGAGTCCTAACCACTGGACCACCGGGGAAGTCCCACCCCCACAACTCTTGTGAATCTGAAGTTCTATACCTATTAACTCTCCTGTTCACCTCCCCTCAGCCCCTGGAAACCATCGTTCTACTTTTTGTCTCTATGGTTTTGACTAATCTAAGTACTTGGTATAAGTGCTTATAAGAATTATACAGTACTTGTCTTTTTGTGATTGGTTTATTTCACTTAGCAGAATGTCCTCAAGGTTCATCCAAGTTGTAACATGTGTCAGAATTTCCTTTCTTTTTATGTATCTATTATTACTATTTAAAAAAAATTTTTTGCCACACTTTGCAGCATGCAGGATCT

TATTAATAGTTCCCTGACCAGGGATGGAACCCACTCCCTCTGCAGGAGAAGCGTGGAGTCTTAACCACTGGACTGCCAGGTGAGTCCCTAGACATAGCTTCTTTTTAATTAATTTTTAAATTCTTGGCTGTGCAGGGTCTCCCTTGCTTTGCGCAGGCTTTCTTTGGTTGCAGTGAGTGGGGACGACTCTTCATTATGGTGATCAGGGTTCTCATTGCTGTGGTTTCTCTTTTTGTGGAGAACAGGCTGTGGGGCACACAGGCTCAGCAGTTGTGGCGCACAGGCTTAGCTGCTGGGATCTAACGTGGAATCCTCCTGGACCGTGGATCGAATTCGTGTTTGCTGCATTGGCAGGGGAGATTCGCTGTTGCAACAGGTGGGATGCAGAATCAGATGTGAGAATCCAGCTGTCTTCTGTTCACTGGACATGGAAGAGATGCAAAACTGGAAAACAATGTCTCTCTTTTTATGGAATATTTTTGTTTTGGAAAAGGTGGCTATTTTTCATTAAA

>ENSCHIT00000002293 gene=ENSCHIG00000001641

GATGAATTTGAGTAGACTCAGGGAGCTGGTGATAGACAGGGAAGCCTGGTGTCCTACAGTCCATGGAGTCGCAGTGAGTCAGATACAACTGAACTAGTGAACTAAGCTGAACTGAACACTTTTAACAGCATATAGATGATATGTCTGTCCTTTTATCTGTTACTTTTTCAAAGAAAAAAAATAAGAAGGCAAAAGAATGATACGTGAAAATGTAAACCAAAGAGATGTTTCTATAGTATGGCATCACGGAATTTTTTGAAACTGAAAAATTTTCTAACAGCCAAATGTGTGGATCTTGGGGGTACTTTGGAAAATTCAGGCACTGCAGTTCCTGCAGGAAGTGTGGCCTGCGGATGTCCATTGCATAGCAGAAGCGAGTTTGGAAAAATGCTGACACTCAGCTTCTTTTTAGCAAATTTGCAGGTGGATCTTATGGGTGCACCGGAAAGATAACATCCGAAGGAAGATCAGAAGTCAACAGGTCTTCCCACAATGGAAAAATGATGACACATCTGACATCTTACCTTAATGATTAACTCAGATTGCTTCACCTTTCTCCGTTTAAAAAAAAGCATGGCTGAGCAGAATATTCAGAGTTGGACACAAGTCTACTTTCTCTCCAGCTTTTTGGAGTAAAAGTCTCTCCTTTCTACCAACACTTGCCCCTCAAATATTGGCTTTTGAGCAGAGACCTGCTGGACTGGAGTTCAGTAACAAAAAGAGCTCTCTTGGACAGAGGACTGAGAGACAGGGTTCCAGCTCCCTCTTTCCTACCGCAGTCACTGATTTTCAGTTTCCTTACTTGTCAAACATGGGCTTTGGTTAGCACCGGCTGATTTACAAGGTCTCGTTTCACTGCAAGACTCTGGATTCAGGTCTCATGAAGCTGAATTAAATGTTCTTTTGCTATCATCACAGTTCCCTCCTTCTTCCCTAGGAAATAGAAAACTTCTGCACTTGAGAATACTATCTGACTTCCAAAAAATGTCCATACACTTGTATTTTATAGGTTCTGTTTTAACGAAACAAGCCTACTGTCAACCTGCGAACTCTCACGATTGTTGAAATGCAAAAGCAAAGTGAAAGAGCAAATCATTTGATTATTTTTCTTCGATAGCAAGGAATAAAAGTAACTTTGTCTCTCCTAGTATAGCCATTCCTGGCAATTCATTATATCATGGGGAAAAAAAT

>ENSCHIT00000006977 gene=ENSCHIG00000005237

ATGGGAGTGATGATGAGAAGTCAGCCCCTGGCGGCCAGCCGGGAGAGCGCTCCTGACCTGAGGAGCCGGTGCAGAGTTCAAGCTGTGGAGGCGCTTGGCAAGTCCCAGAGAGCGGAAGTCAAGGAGAGGAGCCTGGAGAGAAGCAGGAAGGCCGCTGAAGAGCTGGCTGTGCCCCCGGATGCACAGTCTCGGAAAGACCCCTGCCCACGTCCTGGGAGCCTCCAAAAGCACATCTGGGTGGTGCGTGCTGCATGAGCGCAGTGGGTGCACATCAGTCGCCCCACGTGGCCAGGAGGCACAGCAGACCCAGAGCACATGGACCGAGGAACAGAAAGCCGCCCGCCAACGGCCAGACAGCTAGAGGAAACGCAGTGCAGATGTTTTGCTCATGCTCTCAAAAGACCAGAAGATTGGTCTCCCTTTGGTGTGCTCAAGACAGGCACTATGTTCCAGAAAGAAGTTATTTATCATCTTAAACTTAGACTCCAGGCCCCGTTCCACTTGACCTCGTTGTAAGATCAAGGGCTGTGTAAGCTGAAGATGAAGAGAAAAAGTTAAAATTTTACATAACCTCCTGCTCATTCTGCCTCTGTAACTCAGCTTGCCCCTTGCAAAGTCTAGATCACGTAGGTCACATAGTCTTAGAAAGTGGGGACTTGGAATACTAGGAACTGGAGTTTATCTCACTCACCCTTGTCCTGCTCTTTGCAATTGCCCAGCCCCTGCAATCCTGCTTAATCATGCCTGTCCGTGTAAAGGTCAGGCAACCCCCTCCCCATCGTGACTGCTGTTCCCGCATGTGCGAAACCCCTTAGTTTAAACCAGCCTGTTAGCAGCTAGTGTTGCCCACTACAGTCTGTCTATAAAAACTCTGTAACCCCTTTGTTCCGGGCTTAGAGCTTGGAGTGTT

AACTCCTCTGGGCTGGCTGGTGTAATAAACCTGAGTTTTCCAACTCTCCAAGTGTGGTGCTTCGTTTCTCGAGTACTGGTTTCTGTGACAAAGAGAGAGACATATTGATGAGAATCTGATCCTAACATCTGAGCAGAGCCACGCGAGTTGGGAACCAGGTCAGTTCGACAGACATTCACTGAATGTCTATGAGCACATGCTGAGTCAGATCTGCACAGAATCAGATAAGACCAGGGCCCTGCCCGAAGGGACTTCACCTCCAGGAGATGGGCACATAATCTTGAGATTGGTGCTGACAGAGACCCATGCCCACGTGTCAGGGGAGGAATCCAGGTGTTTAAGGAAGGCTTCCTGGAGAGTGTCATGCCTGAGTACATACCTGAAAGATGAGTGCGACCTGGCCTGATGGTGATGAATGAGGGAAGCATTCCAGGAAGAGGCAGGATTGTAAATAAAGCGATGAACATGAGAACTGCAGGCAGCCTGCAGGAGCACAGCGCCTGAGGCGGAGAAATACTGGCCTGGAAAGGTACAGACAGGTCAGAGCAGAAGGAGGCAAGGCCACATTTGAATTTAGAGTTCTGTGGAGACTGGACTGCAGCAGGTATTAATAGTTTCAGCAGAG
